# Collagen vitrigel promotes hepatocytic differentiation of induced pluripotent stem cells into functional hepatocyte-like cells

**DOI:** 10.1242/bio.042192

**Published:** 2019-06-10

**Authors:** Shun Nakai, Ima Shibata, Takahiro Shitamichi, Hiroyuki Yamaguchi, Nobuyuki Takagi, Tomoaki Inoue, Toshito Nakagawa, Jumpei Kiyokawa, Satoshi Wakabayashi, Tomoya Miyoshi, Eriko Higashi, Seiichi Ishida, Nobuaki Shiraki, Shoen Kume

**Affiliations:** 1 School of Life Science and Technology, Tokyo Institute of Technology, 4259-B-25 Nagatsuta-cho, Midori-ku, Yokohama, Kanagawa 226-8501, Japan; 2 Isehara Research Laboratory, Technology and Development Division, Kanto Chemical Co., Inc., 21 Suzukawa, Isehara, Kanagawa 259-1146, Japan; 3 Technology and Development Division, Kanto Chemical Co., Inc., 2-1, Nihonbashi Muromachi 2-chome, Chuo-ku, Tokyo 103-0022, Japan; 4 Research Division, Chugai Pharmaceutical Co. Ltd, 1-135 Komakado, Gotemba, Shizuoka 412-8513, Japan; 5 Pharmacokinetics and Metabolism, Drug Safety and Pharmacokinetics Laboratories, Taisho Pharmaceutical Co., Ltd, 1-403 Yoshino-cho, Saitama-shi, Saitama 330-8530, Japan; 6 Toxicology and Pharmacokinetics Laboratories, Pharmaceutical Research Laboratories, Toray Industries, Inc., 6-10-1 Tebiro, Kamakura, Kanagawa 248-8555, Japan; 7 Division of Pharmacology, National Institute of Health Science, 3-25-26 Tonomati, Kawasaki 210-9501, Japan

**Keywords:** Hepatocytic differentiation, *In vitro* differentiation, Induced pluripotent stem cells

## Abstract

Differentiation of stem cells to hepatocytes provides an unlimited supply of human hepatocytes and therefore has been vigorously studied. However, to date, the stem cell-derived hepatocytes were suggested to be of immature features. To obtain matured hepatocytes from stem cells, we tested the effect of culturing human-induced pluripotent stem (hiPS) cell-derived endoderm cells on collagen vitrigel membrane and compared with our previous reported nanofiber matrix. We cultured hiPS cell-derived endoderm cells on a collagen vitrigel membrane and examined the expression profiles, and tested the activity of metabolic enzymes. Gene expression profile analysis of hepatocytic differentiation markers revealed that upon culture on collagen vitrigel membrane, immature markers of *AFP* decreased, with a concomitant increase in the expression of mature hepatocyte transcription factors and mature hepatocyte markers such as *ALB*, *ASGR1*. Mature markers involved in liver functions, such as transporters, cytochrome P450 enzymes and phase II metabolic enzymes were also upregulated. We observed the upregulation of the liver markers for at least 2 weeks. Gene array profiling analysis revealed that hiPS cell-derived hepatocyte-like cells (hiPS-hep) resemble those of the primary hepatocytes. Functions of the CYP enzyme activities were tested in multi-institution and all revealed high CYP1A, CYP2C19, CYP2D6, CYP3A activity, which could be maintained for at least 2 weeks in culture. Taken together, the present approach identified that collagen vitrigel membrane provides a suitable environment for the generation of hepatocytes from hiPS cells that resemble many characteristics of primary human hepatocytes.

## Abstract (precis)

**Summary:** We found that collagen vitrigel membrane used as scaffold potentiates differentiation of human induced pluripotent stem cells to differentiate into mature hepatocyte-like cells that exhibit mature functions of the hepatocytes.

## INTRODUCTION

The human liver is an important organ that performs many complex functions in metabolism and biosynthesis, the storage of essential nutrients, and biotransformation, including detoxification and bioactivation of drugs. Primary human hepatocytes are the gold standard for studies for the pharmacological assay to predict drug transport and clearance, drug–drug interaction and hepatotoxicity (Godoy et al., 2013). However, the supplies of primary human hepatocytes are limited and there is considerable donor-derived variability (Guo et al., 2011). Human embryonic stem (hES) cells or human-induced pluripotent stem cells (hiPS cells) are capable of undergoing unlimited self-renewal, retaining their potential to differentiate into any somatic cell types (Takahashi et al., 2007; Thomson et al., 1998). Upon addition of suitable growth factors, the hES/hiPS cells could recapitulate normal developmental processes, and give rise to cells deriving from the three germ layer, and therefore supposed to serve as an alternative resource for studies of developmental biology, drug discovery, toxicology, as well as disease modeling and cell replacement therapy (Godoy et al., 2013; Shi et al., 2017).

Through our present knowledge of developmental biology, efficient induction of hepatocytic lineage cells has been established. Fibroblast growth factor (FGF) and bone morphogenetic protein (BMP) activate dynamic signaling pathways for the specification of liver lineages (Gouon-Evans et al., 2006; Jung, 1999; Katsumoto et al., 2010; Rossi et al., 2001; Shin and Monga, 2013; Si-Tayeb et al., 2010a). Hepatocyte growth factor (HGF), dexamethasone and oncostatin M have been employed for induction of hepatocyte maturation (Hannan et al., 2013; Kamiya et al., 1999; Schmidt et al., 1995; Si-Tayeb et al., 2010b). However, hepatocytes generated from pluripotent stem cells showed immature profiles of protein expression and cytochrome P450 (CYP) enzyme activities, and are suggested to better mimic fetal rather than adult hepatocytes (Baxter et al., 2015; Si-Tayeb et al., 2010b). Although the signaling events that potentiate the final maturation processes are not fully understood, recent reports suggested that hepatocytes derived from hES/hiPS cells offer a good *in vitro* human model in metabolic testing of the drugs and drug toxicity for predicting liver toxicity (Gao and Liu, 2017; Kim et al., 2017; Lu et al., 2015; Takayama et al., 2013).

To promote differentiation of the directed hepatocytic differentiation, we examined the role of extracellular matrices (ECM) and scaffolds. We previously reported that culturing hES/hiPS cells on a mesonephric cell line, M15, with specific growth factors resulted in an efficient induction of endoderm, which then gave rise to various endoderm-derived tissues, such as liver, pancreas or intestine (Ogaki et al., 2013; Shiraki et al., 2008a,b; Umeda et al., 2013). M15 cells expressed basement membrane components, including *lama5*, which played an important role in lineage specification of the hES/hiPS cells from endoderm. The importance of the basement membrane components was shown by using a synthesized basement membrane substratum, or a synthetic nanofiber matrix (Higuchi et al., 2010; Shiraki et al., 2011; Yamazoe et al., 2013). The nanofiber matrices promoted *in vitro* hepatocytic differentiation of hES/hiPS cells and were more potent than normal plates pre-coated with other matrices. The hiPS cell-derived hepatocytes grown on the nanofiber matrix expressed higher levels of differentiation markers. However, the expression levels of transporters or metabolic enzymes still required to be improved.

A collagen vitrigel (CV) membrane is composed of high-density collagen fibrils equivalent to connective tissues *in vivo* and possesses excellent transparency and permeability of protein with high molecular weight, which has been described to be utilized as a cell culture scaffolds (Takezawa et al., 2007; Wang and Takezawa, 2005). Collagen vitrigel membrane was reported to facilitate three dimensional formation of human corneal epithelial cells, and was useful to induce HepG2 cells to form hepatic structures that exhibited CYP3A4 activity and supported liver-specific functions of hepatocytes in long-term culture (Oshikata-Miyazaki and Takezawa, 2016; Watari et al., 2018; Yamaguchi et al., 2013, 2016).

We previously reported that the undifferentiated hES/hiPS cells exhibit a high dependence of methionine metabolism and deprivation of methionine for a long term as long as 48 h could trigger apoptosis of the undifferentiated cells, whereas a transient deprival of methionine for 5–7 h could render the cells at a poised state for differentiation. With this finding, we have shown that a short methionine deprival for 5 h with undifferentiated human hES/hiPS cells followed by application of differentiation signals led to a potentiated differentiation (Shiraki et al., 2014).

Here, we tested the effect of culturing the hiPS cell-derived endoderm on a CV membrane and induced hepatocytic differentiation. We examined the expression profiles and activities of the induced hepatocyte-like cells derived from hiPS cells.

## RESULTS

### Hepatocytic differentiation of hiPS cells on collagen vitrigel membranes

Previously, we showed that a synthetic nanofiber matrix promotes *in vitro* hepatocytic differentiation of pluripotent stem cells. Here, to search for other matrices that could potentiate hepatocytic differentiation, we tested the effect of collagen vitrigel (CV) membrane in potentiating the differentiation of hiPS cells to hepatocytes, in comparison with the nanofiber matrix.

**Fig. 1.**
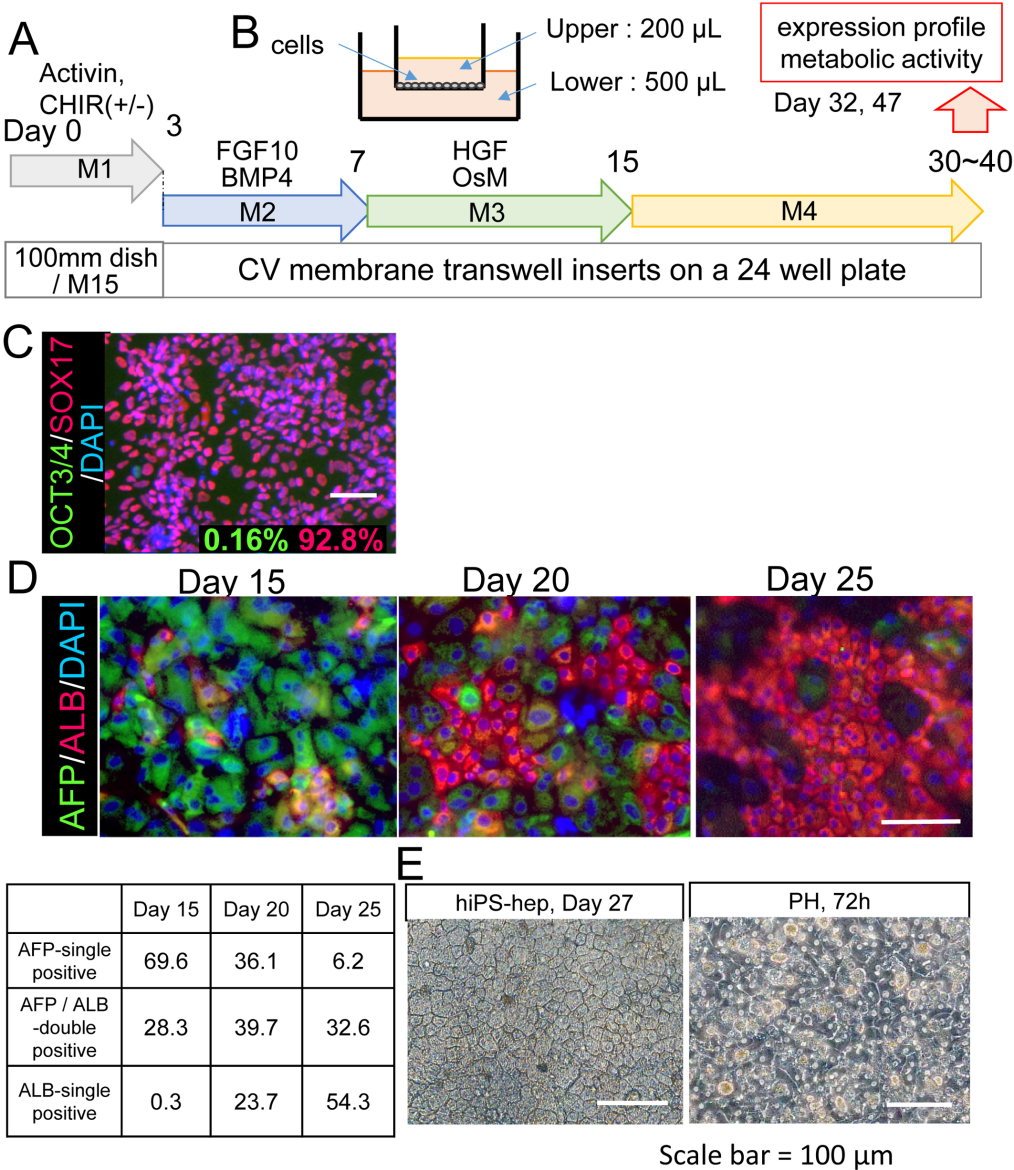
**Differentiation of hiPS cells into the hepatocyte lineage on collagen vitrogel membranes.** (A,B) Schematic diagram of the differentiation of hiPS cells into hepatocytes. Undifferentiated hiPS cells (treated with methionine deprival for 5 h) were differentiated into endoderm on M15 cells. hiPS-derived endoderm cells on day 3 were dissociated and frozen until use. Upon differentiation, the frozen endoderm was thawed and seeded onto the CV membrane transwell inserts (B), supplied with media 200 μl in the upper chamber and 500 μl in the lower chamber. Media used for each step of differentiation are as indicated. hiPS-derived hepatocytes (hiPS-hep) were subjected to expression profile analysis or metabolic activity tests between differentiation day 30 (D30) and 47 (D47). (C) Immunochemical analysis of hiPS-derived endoderm cells. Green: OCT3/4 (0.16±0.02%), red: SOX17 (92.8±0.14%). (D) Immunochemical analysis of differentiated hiPS cells on D15, D20 and D25 for alpha-fetoprotein (AFP, green), albumin (ALB, red) with nuclear counterstaining (DAPI, blue). Percentages for AFP-single positive, ALB-single positive and AFP/ALB-double positive cells are shown in the table. (E) A representative phase contrast image of hiPS-hep differentiated on the CV membranes on D27 demonstrated polygonal morphologies that resembled hepatocytes. As a control, a representative phase contrast image of primary hepatocytes (PH) grown on collagen-coated plates for 72 h after freeze-thawed is also shown. Scale bars: 100 μm.

In the present study, we initially used ChiPS18, a hiPS cell line, which was reported to show good hepatocytic differentiation efficiency (Asplund et al., 2015). We adopted our culture procedure for undifferentiated hiPS cells as described in the Materials and Methods. We first prepared hiPS cell-derived endoderm cells (hiPS-endo) by culturing ChiPS18 on M15 feeders for 3 days and harvested the hiPS-endo on day 3 (D3) (Fig. 1A,B). The hiPS-endo were stocked as frozen cells and were confirmed to exhibit 92.8±0.14% SOX17 and 0.16±0.02% OCT3/4 positivity upon freezing and thawing and cultured overnight (Fig. 1C).

To perform hepatocytic differentiation, the hiPS-endo were freeze-thawed and plated onto the CV membrane. The hiPS-endo were differentiated into hepatocytic fates, by culturing with sequential changes of media, first with M2 media on day 3–7, changed to M3 media on day 5–15, and then to M4 media on day 15–30 or up to day 47. In our preliminary tests with different thicknesses of CV membranes, we assayed for the expression of several hepatic differentiation markers by real-time RT-PCR. We found that their expression was potentiated when hiPS-endo were seeded onto a 1× thickness compared to 0.5× or 2×, 10× fold thickness of the CV membranes and cultured until day 32 (Fig. S1). We then focused our studies using the present form of the CV membrane and compared with those cultured on the nanofiber matrix pre-coated with Matrigel (Yamazoe et al., 2013) or normal tissue culture 2D plates pre-coated with SynthemaxII as a control.

Immunocytochemical analysis of the hiPS cell-derived differentiated cells revealed that hiPS cell-derived cells turned into alpha-fetoprotein-single positive (AFP+) hepatic progenitor cells (69.6%), and 28.3% AFP/albumin (ALB)-double+ hepatoblasts on day 15. AFP expression decreased to 36.1% and ALB-single+ cells increased to 23.7% on day 20. Then, on day 25 AFP-single+ cells were approximately 6.2%, whereas ALB-single+cells were 54.3% (Fig. 1D). Polygonal morphology of the hiPS-derived cells grown on the CV membrane resembled the morphology of human primary hepatocytes (Fig. 1E).

### hiPS cell-derived hepatocytes grown on CV membrane showed a more mature expression profile than those grown on nanofiber substrate

**Fig. 2.**
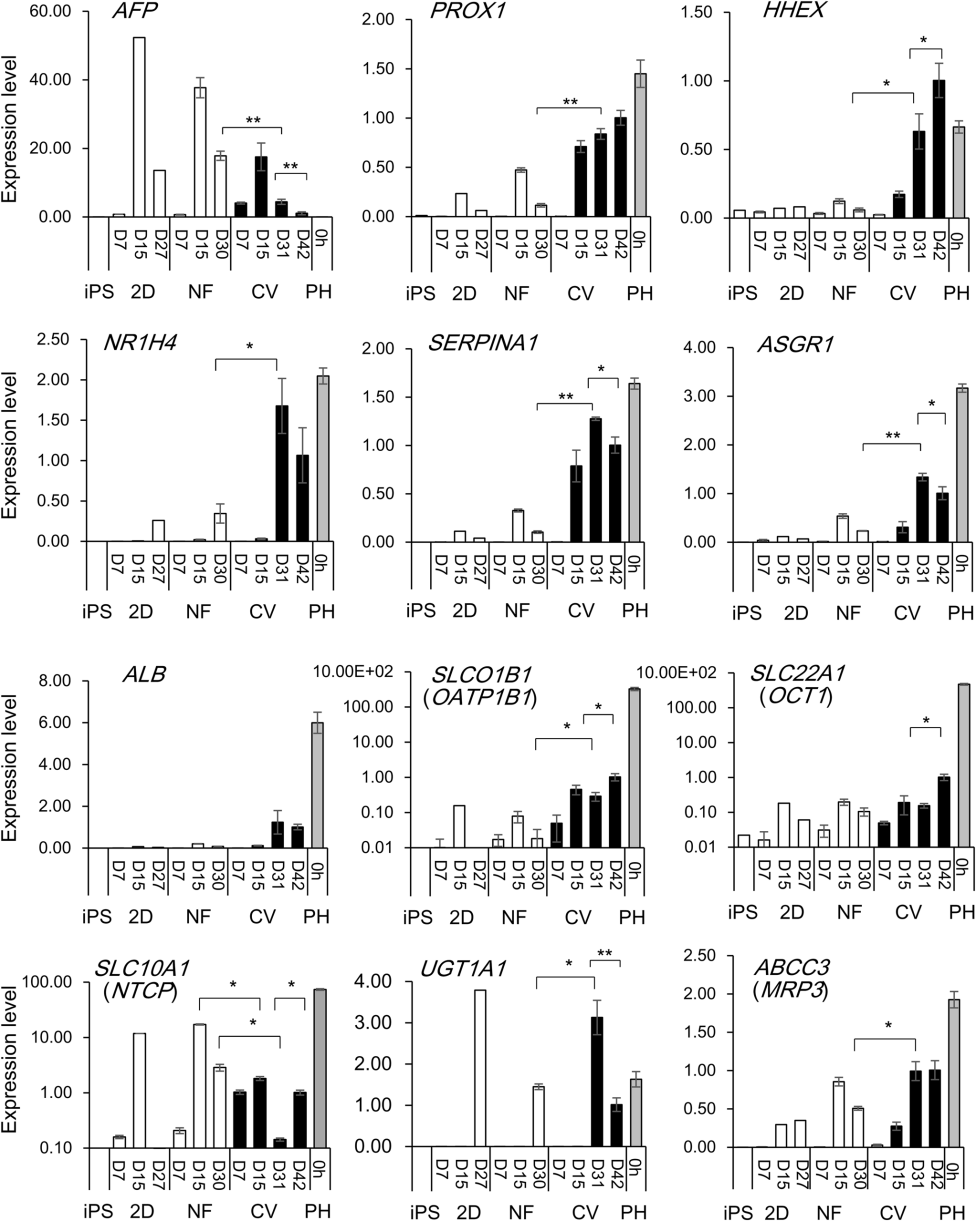
**Expression of hepatocytic markers in differentiated hiPS cells on the collagen vitrogel membranes, nanofiber matrices or 2D plates.** Expression levels of various gene transcripts quantified by real-time PCR in undifferentiated hiPS cells (iPS) or D7, D15, D27, D30 or D42 differentiated hiPS cells on 2D plates pre-coated with SynthemaxII (2D), nanofiber (pre-coated with Matrigel; NF) or the collagen vitrigel (CV) membrane are shown. Primary hepatocytes (PH, 0 h; no pre-culture) were used as a reference. For differentiated hiPS cells, values represent means±s.d. (*n*=3). Relative values are shown, with the value of D42 hiPS cells grown on CV=1. Significance shown as *P<0.05 or **P<0.01, by two tailed Student's *t*-test, compared between hiPS-hep grown on CV (D31) versus NF (D30); or CV (D31) versus CV (D42).

To characterize the hiPS-derived hepatocyte-like cells (hiPS-hep), which are differentiated for more than 25 days in our culture, we performed real-time RT-PCR to examine the expression of hepatocytic molecular markers using a primer set consisting of 88 known genes related to hepatic differentiation and eight housekeeping genes (Fig. 2; for the full list of markers, refer to Table S1). The results were compared between hiPS-hep grown on the CV membrane and those grown on the nanofiber matrix, which we previously reported to be a potent substrate for supporting hepatocytic differentiation. As a control, RNA extracted from cryopreserved primary hepatocytes (PH) without pre-incubation was used (PH 0 h). The expression of *AFP*, an immature marker, almost disappeared in PH and was observed to increase on differentiation day 15 (D15) but then decreased with increasing days of differentiation in hiPS-derived differentiated cells grown on the CV membrane, compared to those on nanofiber or the 2D environment. Transcription factors such as *Prospero homeobox 1* (*PROX1*) or *Hematopoietically expressed homeobox* (*HHEX*), which are known to promote the expression of the mature markers, are upregulated in hiPS-derived differentiated cells grown on the CV membrane compared to those grown on the nanofiber matrix. Mature markers, such as *nuclear receptor subfamily 1H4* (*NR1H4*; encodes farnesoid X receptor; FXR), *SERPINA1* (encodes alpha 1-antitrypsin; AAT) or *asialoglycoprotein receptor 1* (*ASGR1*) (Peters et al., 2016), which are highly expressed in primary hepatocytes, were highly expressed in hiPS-hep grown on the CV membrane, compared to those grown on the nanofiber matrix or 2D plates. The expression of *ALB* was approximately 1/6-fold, *ASGR1* approximately 1/3-fold of the expression in PH. Taken together, these transcription profiles suggest that hiPS-hep grown on the CV membrane represent more mature hepatocytes than those grown on the nanofiber matrix.

Uptake transporters such as *Solute carrier organic anion transporter1B1* (*SLCO1B1* or *OATP1B1*) and *SLC22A1* (or *OCT1*; *organic cation transporter1*) were expressed in hiPS-hep grown on CV at a higher level than those grown on the nanofiber matrix. *Solute carrier family 10 member 1* (*SLC10A1* or *NTCP*; *sodium/taurocholate cotransporting polypeptide*) expression was higher in those grown on the nanofiber matrix compared to the CV on day 31 but increased later in those grown on the CV on day 42 of differentiation. Phase II metabolizing enzymes, such as *UGT1A1* (*UDP glucuronosyltransferase 1alpha 1*), and an excrete transporter *ATP-binding cassette, sub-family C3* (*ABCC3* or *MRP3*) were expressed in hiPS-hep grown on CV at a higher level than those grown on the nanofiber matrix (Fig. 2). These transcription profiles suggest an overall more mature feature of the hiPS-hep which could be obtained by culturing on the CV membranes.

**Fig. 3.**
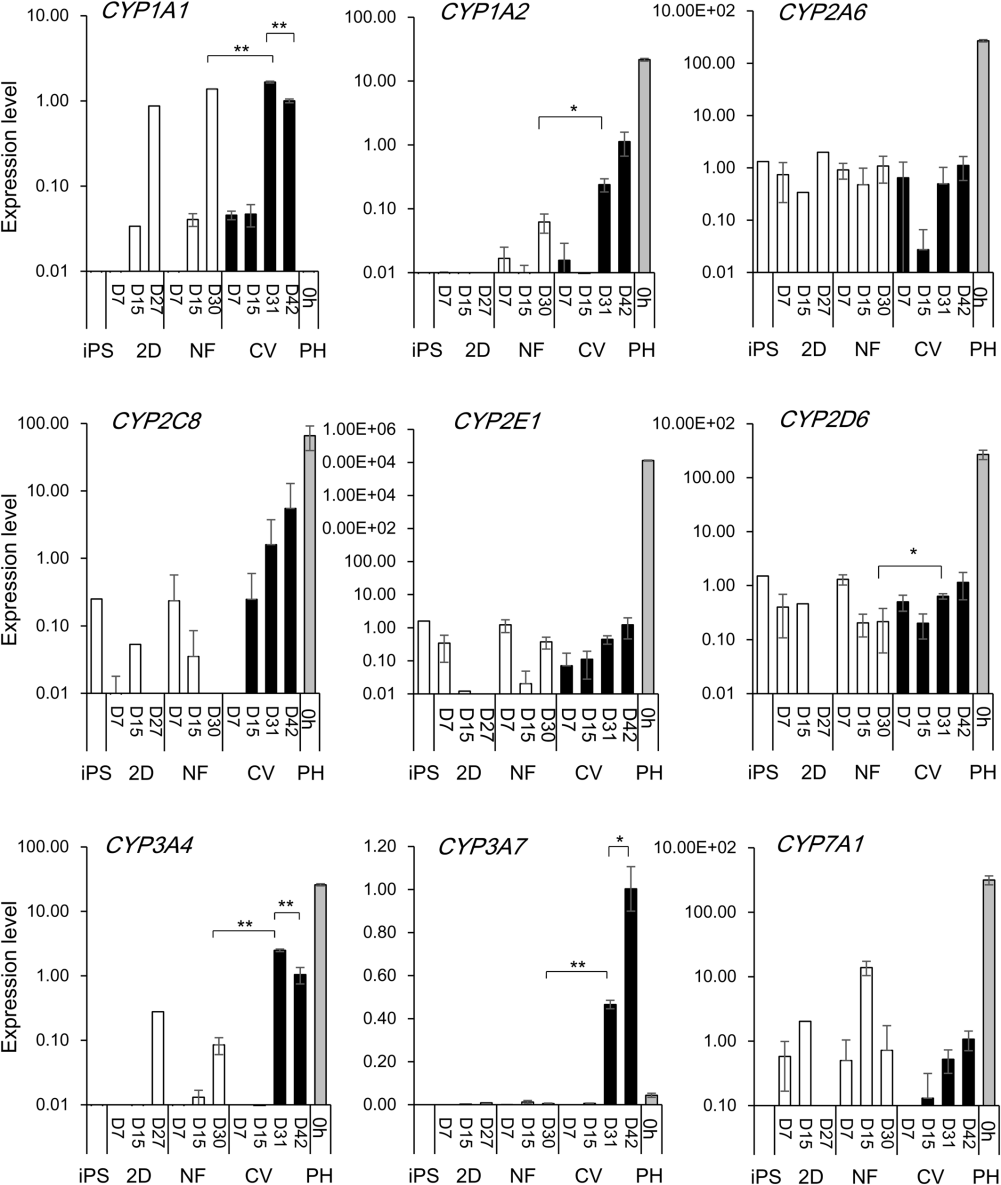
**Expression of CYP metabolizing enzyme genes in differentiated hiPS cells on the collagen vitrogel or nanofiber matrices or 2D plates.** Expression levels of various metabolizing enzymes were quantified by real-time PCR in undifferentiated hiPS cells (iPS) or hiPS-hep, grown on tissue culture plates pre-coated with SynthemaxII (2D), nanofiber (pre-coated with Matrigel; NF) or the CV membranes. Primary hepatocytes (PH, 0 h) were used as a reference. For differentiated hiPS cells, values represent means±s.d. (*n*=3). Relative values are shown, with the value of D42 hiPS cells grown on the CV=1. *P<0.05 or **P<0.01, by two tailed Student's *t*-test, compared between hiPS-hep grown on CV (D31) versus NF (D30); or CV (D31) versus CV (D42).

We then analyzed the expression profiles of metabolic enzymes. Metabolic enzymes, such as *cytochrome P450* (*CYP*) *1A1*, *CYP1A2*, *CYP2D6*, *CYP3A4* and *CYP3A7*, were expressed at higher levels in hiPS-hep grown on the CV compared to those grown on the nanofiber (Fig. 3). Other CYP enzymes, such as *CYP2A6*, *CYP2C8*, *CYP2E1* and *CYP7A1* (involved in cholesterol metabolism) showed expression levels similar between hiPS-hep grown on the CV and the nanofiber. Whereas *CYP3A7*, a fetal CYP enzyme was expressed at a high level in hiPS-hep grown on the CV. *CYP1A1* expression was higher than the PH (0 h), *CYP3A4* was approximately 1/50-fold of that in the PH (0 h). Many of the *CYP* enzyme expressions were observed at a substantial level at D31 and D42, which suggests that the functionality of the cells could be maintained for approximately 11 days.

**Fig. 4.**
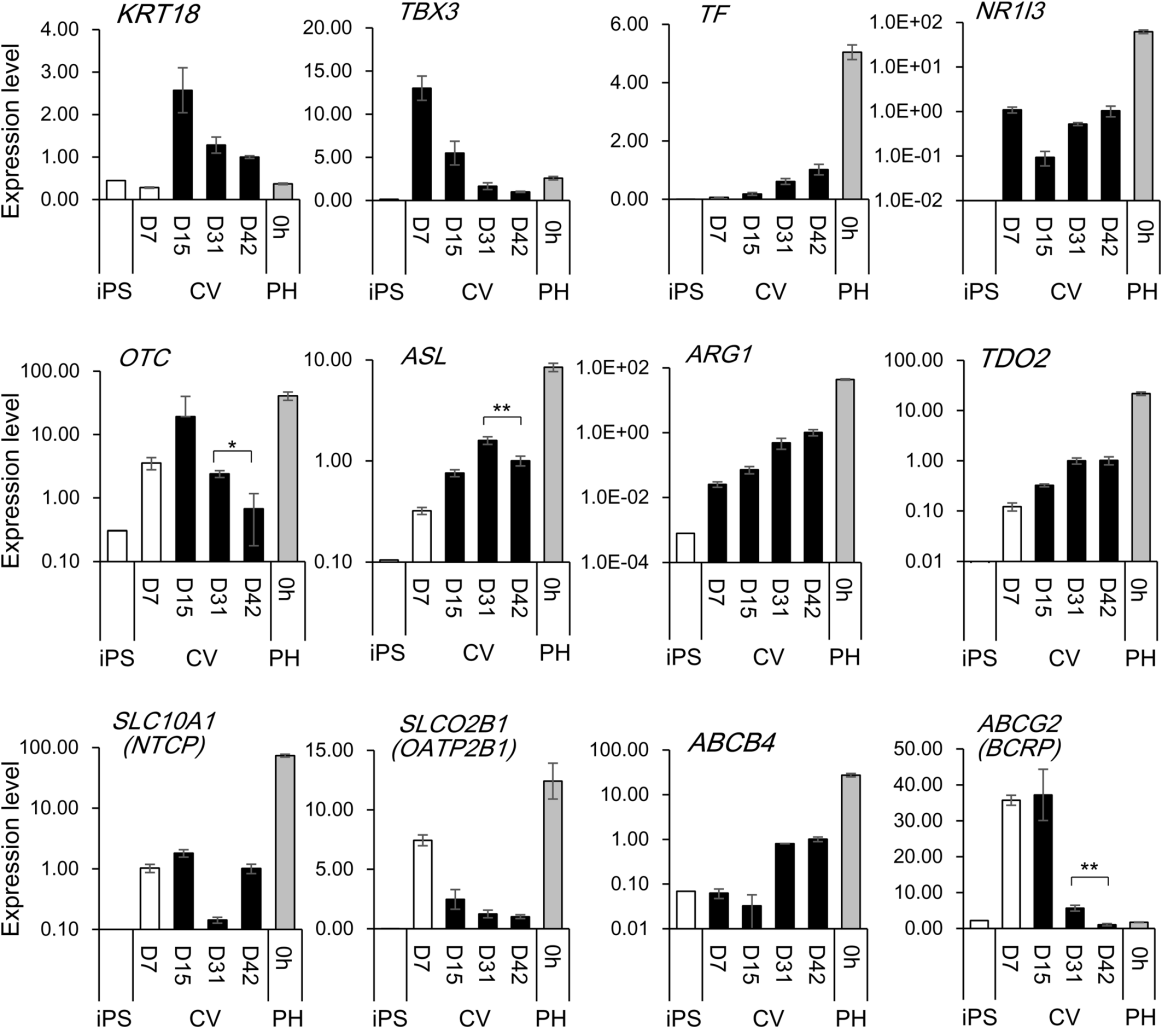
**Hepatic genes expression in undifferentiated hiPS cells or differentiated hiPS cells on the CV membranes.** Expression levels of various genes associated with hepatic differentiation or matured function of the hepatocytes were quantified by real-time PCR in undifferentiated hiPS cells (iPS) or hiPS-hep, grown on the CV membranes. Primary hepatocytes (PH, 0 h) was used as a reference. For differentiated hiPS cells, values represent means±s.d. (*n*=3). Relative values are shown, with the value of D42 hiPS cells grown on CV as 1. *P<0.05 or **P<0.01, by two tailed Student's *t*-test, compared between hiPS-hep grown on CV D31 versus D42.

Other hepatic markers, for example, *keratin 18* (*KRT18*) and *T-box 3* (*TBX3*) were expressed in earlier hiPS-derived cells but reduced with increasing days of differentiation (Fig. 4). *Transferrin* (*TF*) or *nuclear receptor 1I3* (*NR1I3*; or *constitutive androstane nuclear receptor*, *CAR*) expressions were also detected. Other mature liver markers, such as the urea cycle enzymes *ornithine carbamoyltransferase* (*OTC*), *argininosuccinate lyase* (*ASL*), *arginase* (*ARG1*), as well as *tryptophan 2,3-dioxygenase* (*TDO2*), an enzyme involved in tryptophan metabolism, were expressed. Uptake transporter *solute carrier family 10A1* (*sodium/bile acid cotransporter family*; *SLC10A1* or *NTC*P), *SLCO2B1* (*OATP2B1*), excretion transporters *ABCB4* (*MDR3*), *ABCG2* (*BCRP*) were expressed in hiPS-hep.

Many of the mature markers of the hepatocytes are upregulated in hiPS-hep grown on CV than nanofiber matrix. Importantly, many of the matured marker expressions could be observed at substantial levels in hiPS-hep on day 31 or 42, thereby suggesting that hiPS-hep are functional and could be maintained for more than 10 days.

### Principal component analysis revealed that hiPS-derived hepatocytic cells grown on CV gradually diverged from those grown on normal 2D or nanofiber and expressed a higher level of matured hepatocyte markers

**Fig. 5.**
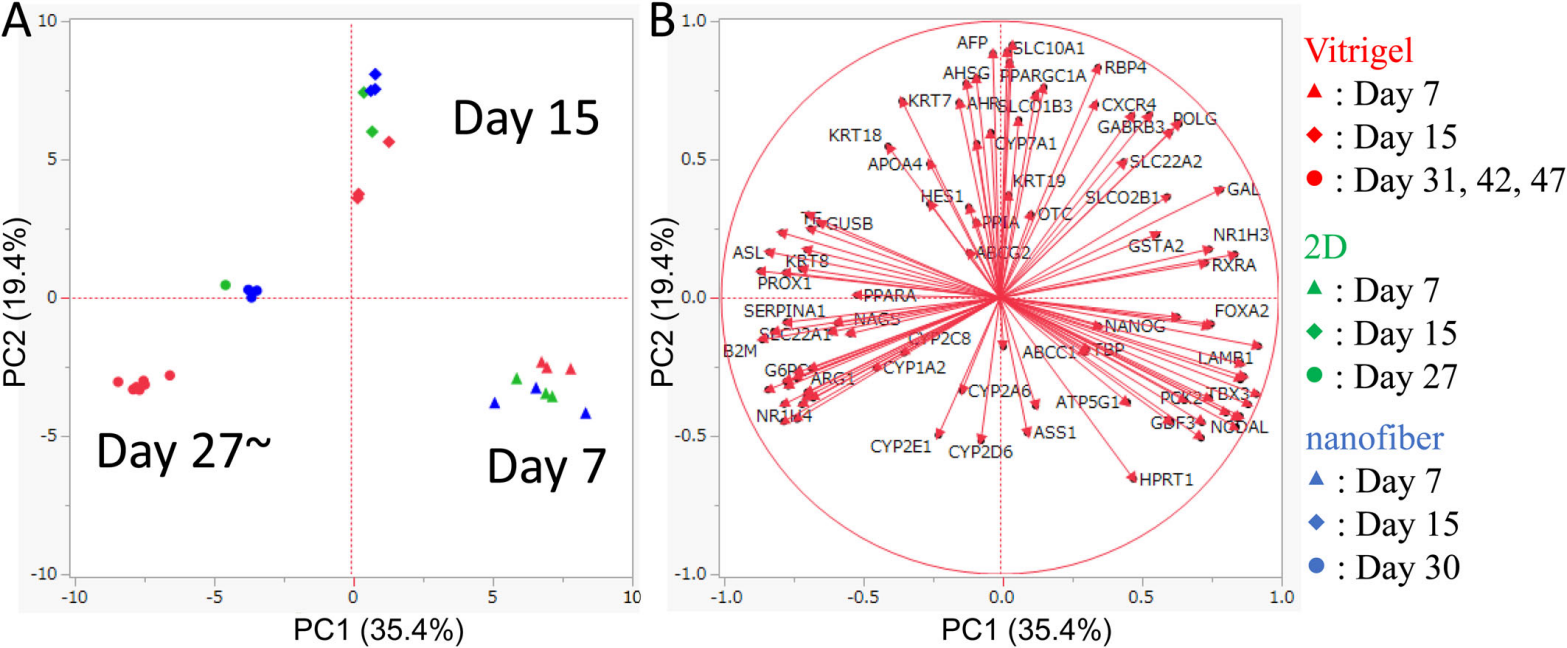
**PCA of gene expression revealed the late hiPS-hep cells grown on CV are distinct from nanofiber or 2D environment.** PCA identified a trend that hiPS-derived hepatocytes grown on vitrigel at late stages showed different a hepatocytic gene expression profile from those grown on 2D or nanofiber (NF) plates. (A) PCA of gene expression profile. Expression data of 96 genes using PrimerArray were analyzed. Each sample was mapped to a two-dimensional plot along with its expression levels of genes of significance for PC1 and PC2 axes to give the largest variance. (B) Data obtained from hiPS-derived differentiated cells grown on CV membrane (red), 2D (green), or nanofiber (blue), at differentiation day 7 (triangles), day 15 (diamonds), day 27∼47 (circles) are shown. Each gene is plotted in accordance with their values of PC1 and PC2 axes.

To obtain insights into the expression profile of the hiPS-derived differentiated cells, Principal Component Analysis (PCA) of the above results (Figs 2–4) using PrimerArray gene expression was performed. PCA with the highest variance revealed the following features: day 7, day 15 and day 27 or later hiPS-derived hepatocytic cells grown on 2D, the nanofiber or the CV fall in three distinct clusters, within which hiPS-hep on day 27 or later mapped away from hiPS cells grown on 2D or the nanofiber (Fig. 5A). Although there are some differences in the expression of individual markers among hiPS-hep on day 31, 42 and 47, the difference is smaller than those on day 15 or earlier hiPS-derived differentiated cells.

Genes that contributed to the PCA analysis are shown in Fig. 5B, and are projected onto positions coordinated with their contributions to the PC1 or PC2 axes (Fig. 5B). For example, the genes specifically expressed in day 7 cell clusters are genes such as *FOXA2*, *TBX3*, *NODAL*, *LAMB1*, which are associated with endoderm differentiation. The genes that are specifically expressed in day 15 cell clusters are genes such as *AFP*, *KRT18*, *CYP7A1*, *APOA4*, which are associated with early hepatic differentiation. Genes, such as *SERPINA1*, *SLC22A1*, *NR1H4*, *CYP1A2*, which are associated with the maturation of hepatocytes, are specifically expressed in cell clusters on day 27 or later.

The results, therefore, indicate that the hiPS-derived differentiated cells grown on the CV membrane showed distinct gene expression profiles, which seem to resemble mature hepatocytes.

### Functional analysis of the metabolic enzyme activities of the hiPS-derived hepatocytes

**Fig. 6.**
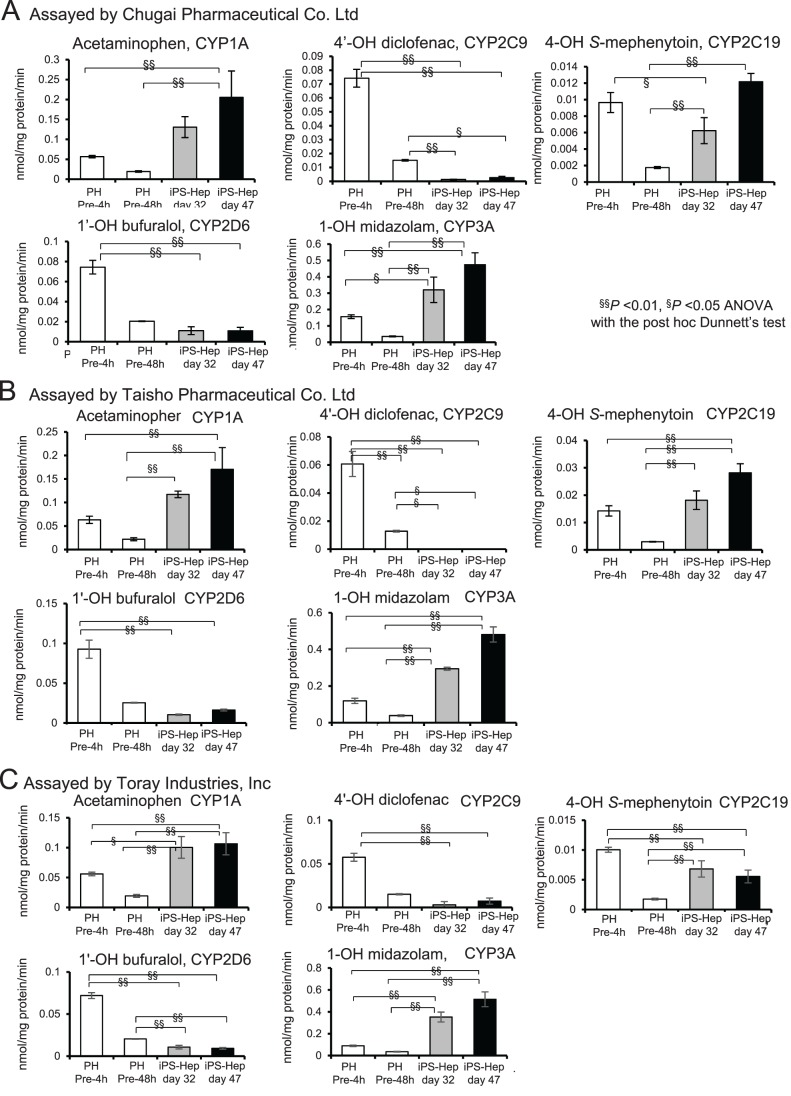
**CYP activities in hiPS-derived hepatocytes on D32 and D47 of differentiation.** (A–C) CYP1A, CYP2C9, CYP2C19, CYP2D6, CYP3A activities in hepatocyte cultures derived from hiPS (iPS) cells after 32 days (gray bars), or 47 days (black bars) of differentiation were determined by LC/MS/MS, normalized to the protein content per well per min using the values obtained at 2 h. Cryopreserved primary hepatocytes pre-cultured for 4 h (pre-4 h) or 48 h (pre-48 h) (open bars) are used as references. Values represent means±s.d. (*n*=3). The significance of differences is shown as ^§^*P*<0.05 or ^§§^*P*<0.01. Compared between data groups obtained with PH pre-4 h or PH pre-48 h, with those obtained from hiPS-heps by one-way ANOVA with the post-hoc Dunnett's test. (A) Assayed by Chugai Pharmaceutical Co Ltd. (B) Assayed by Taisho Pharmaceutical Co Ltd. (C) Assayed by Toray Industries, Inc.

Since CYP1A2, CYP2C9, CYP2C19, CYP2D6 and CYP3A4 are the main cytochrome P450 enzymes that are involved in drug metabolism in the liver, we examined their enzymatic activities in the hiPS-hep. To assess the function of the metabolic enzymes, we tested by adding their substrates, phenacetin, diclofenac, *S*-mephenytoin, bufuralol and midazolam in a mixture, and measured the amount of their metabolites, acetaminophen, 4′-OH diclofenac, 4-OH mephenytoin, 1′-OH bufuralol and 1-OH midazolam, respectively, by LC/MS/MS. In this set of experiments, we used primary hepatocytes pre-cultured for 4 h (pre-4 h) and 48 h (pre-48 h) as positive controls. To validate the CYP enzyme activities of the hiPS-hep cells, the activities of the hiPS-hep cells generated in the same institute were assessed in multi-institutions: in Chugai Pharmaceutical Co. Ltd. (Fig. 6A), Taisho Pharmaceutical Co. Ltd. (Fig. 6B) and Toray Industries Inc. (Fig. 6C), respectively. hiPS cells were differentiated and assayed for the function of the metabolic enzymes three times in Tokyo Institute of Technology, then the frozen reaction mixtures were sent out for LC/MS/MS measurements in the above three different institutions. The results of CYP enzyme activities obtained were similar among multi-institutions, thus confirming the reproducibility of the assays for the functions of the derived hiPS-hep cells.

High levels of metabolites were detected as results of CYP1A, CYP 2C19, CYP 2D6, and CYP3A activities in the hiPS-heps. Metabolite generated by CYP1A or CYP3A activity was approximately 0.1∼0.2 or 0.5 nmol/mg protein/min, respectively, in hiPS-hep on day 47. CYP1A activities on D32 were similar to primary hepatocytes cultured for 4 h (PH pre-4 h), but became significantly higher on D47. CYP2C9 activity in D32 or D47 hiPS-hep was detected, albeit lower than or similar to that of PH pre-48 h. CYP2C19 activities on D32 or D47 were at similar level with that of PH-pre-4 h, but higher than PH-pre-48 h. CYP2D6 activity was approximately similar to PH-pre 48 h. CYP3A activity on D32 and D47 are higher than those of PH pre-4 h. The results assayed by other institutions in Fig. 6B,C are similar to those shown in Fig. 6A, therefore indicating the reliability of the measurement of the hiPS-hep activities. Since *CYP1A2* gene expression on D31 is approximately 1/100-fold, and *CYP1A1* gene expression is 100-fold of that of the PH (0 h), the CYP1A activity might attribute to the high expression of CYP1A1 (Fig. 3). Taken together, our results demonstrate that metabolizing enzyme activities of CYP1A, CYP2C19, CYP2D6 and CYP3A in hiPS-hep grown on a CV could be maintained for more than 2 weeks in culture. The levels of CYP1A, CYP 2C19 and CYP3A on D32 or D47 were almost equivalent to or higher than that of the PH pre-4 h.

### Gene expression profile analysis revealed that hiPS-derived hepatocytes resemble human primary hepatocytes

To assess the hiPS-derived differentiated cells obtained by differentiating on CV membrane, we performed gene array analysis of undifferentiated hiPS cells (day 0; D0), hiPS-endo (D3), hiPS-hepatoblast (D15), and hiPS-hep (D30) as well as cryopreserved PH-pre 48 h, which were harvested after the experiment shown in Fig. 6.

**Fig. 7.**
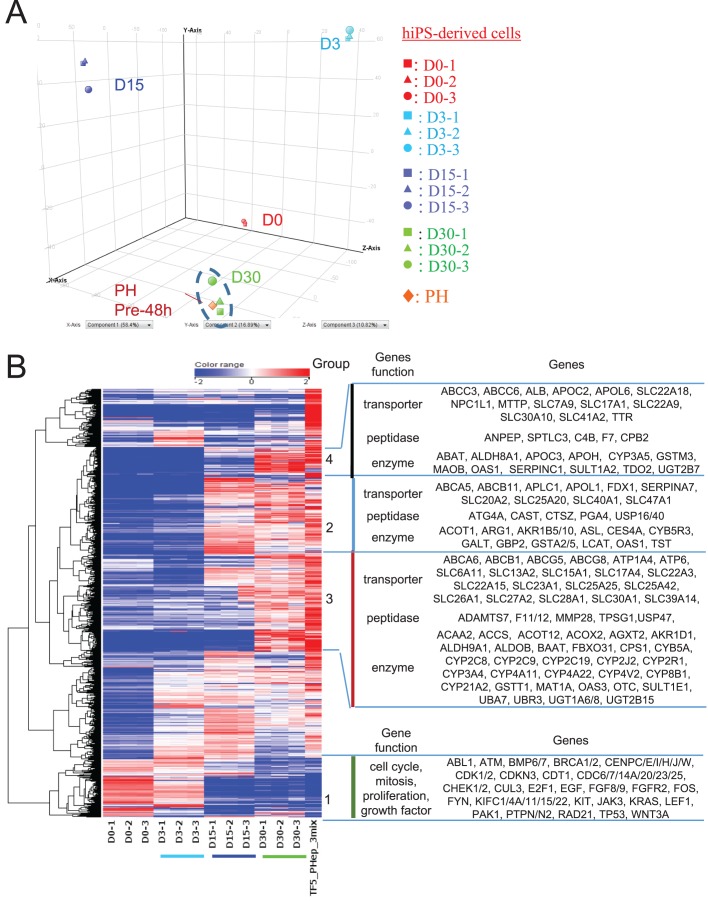
**Comparison of differentiated cells using global gene expression data revealed that D30 hiPS-hep grown on CV resembles primary hepatocytes.** (A) PCA of microarray gene expression profiles. Genes differentially expressed between undifferentiated hiPS cells and D30 hiPS-hep cells are used for the PCA. The undifferentiated hiPS cells, D3, D15, D30 hiPS cells are mapped into a 3D plot to give the largest variance with expression levels of genes of significance for PC1, PC2 and PC3 axes. D0 (red), D3 (light blue), D15 (blue), D30 (green) and primary hepatocytes (PH; orange), are shown. (B) Hierarchical clustering analysis. Normalized expression data are shown in log2 scale and color-coded with the color range shown in the top. The dendrogram on the left shows the clustering of the samples. Representative gene functions and selected genes are indicated for each group.

15,101 genes were extracted to be up- or downregulated between undifferentiated hiPS cells or D30 hiPS-hep cells. PCA of the genes with the highest variance reveals that all three D30 hiPS-hep cells adopt closely neighboring positions with cryopreserved primary hepatocytes, but away from undifferentiated hiPS cells or D3 endoderm cells and D15 hepatoblasts (Fig. 7A). Moreover, within the cluster, cryopreserved hepatocytes were at an even closer position to two of the triplicate hiPS-hep D30 samples. The result, therefore, indicates that the expression profile of hiPS-hep D30 closely resembles that of the PH-pre 48 h.

We then imported the data for Ingenuity Pathway Analysis (IPA). The top 10 gene categories that are up- or downregulated genes in D30 hiPS-hep compared to undifferentiated cells are extracted by IPA, in which genes are categorized by their function annotations. The categories with duplicated functional annotations are omitted. Upregulated genes include those categorized as lipid metabolism, metabolic disease, vitamin and mineral metabolism-related genes. Downregulated genes include those categorized as cancer, cellular development and cell cycle-related genes (Table S2). The top 100 genes that are upregulated in D30 hiPS-hep cells are listed in Table S3. Hierarchical clustering analysis was further conducted with a list of 1633 selected from the above 15,101 genes, with the following biofunctions (according to IPA): cytokine (44 genes), enzyme (1003 genes), peptidase (165 genes), growth factor (37 genes), ligand-dependent nuclear receptor (21 genes), transporter genes (363 genes); and 258 downregulated genes: cell cycle/mitosis (258 genes). The clustering results are shown as a heat map (Fig. 7B). From the heat map, cell cycle-related genes (group 1) were expressed in D0 undifferentiated cells, became gradually decreased in D3 endoderm cells, and further downregulated in D15 hepatoblasts toward D30 hiPS-hep cells. Genes in group 2 are upregulated in D15 immature hepatoblasts, and group 3 and 4 are genes encoding transporters and metabolizing enzymes, which convey important hepatocytic functions, and are upregulated in D30 hiPS-hep cells. CYP enzyme expressions detected in the hiPS-hep by gene profiling analysis are shown as fold expression versus PH-pre 48 h (Fig. S2). The results revealed high *CYP1A1* and *CYP1B1* expression (about 100-fold of the PH pre-48 h) and low *CYP1A2* expression (about 0.01-fold of the PH pre-48 h). *CYP3A4*, *CYP3A5* and *CYP3A7* expressions are 3-fold, 1.1-fold or 5.2-fold of those of PH-pre 48 h, respectively (Fig. S2). Therefore, the CYP3A metabolizing activity observed in Fig. 6 seems to attribute to CYP3A4, CYP3A5 and CYP3A7.

Taken together, the result indicates that D30 hiPS-hep expressed genes that most resemble primary hepatocytes. While cell cycle or mitosis genes that are highly expressed in the undifferentiated or D3 endoderm cells were downregulated, and genes that are related to the mature hepatocytic functions were upregulated in hiPS-hep cells. The gene expression results support our metabolizing enzyme activity results.

Our results, therefore, suggest that the CV membrane is appropriate for supporting hiPS cells to differentiate into hepatocyte-like cells. It is reported that hepatic differentiation potency differs among hES/hiPS cell lines (Kajiwara et al., 2012; Takayama et al., 2014). We also tested other hiPS cell lines, such as ChiPS12 (Asplund et al., 2015) and RPChiPS771 cells (Yoshioka et al., 2013) and found that these cells also differentiated efficiently into hepatocyte-like cells on the CV membrane (S.N., unpublished).

## DISCUSSION

In the current study, we demonstrated the generation of hiPS-hep that exhibit features of the mature hepatocytes, by culturing hiPS-derived endoderm (hiPS-endo) on CV membranes. We referred to the previously published protocol and used BMP4 and FGF10 to induce differentiation into the hepatoblasts, then added with OsM and HGF for induction into the hepatocyte lineage (Hannan et al., 2013). Although FGF10 has been reported to be important for pancreas differentiation (Bhushan et al., 2001), we did not observe the induction of PDX1 or INS expression in the hiPS-derived hepatoblasts or hiPS-hep. The hiPS-endo cultured on the CV membrane gradually downregulated the expression of immature markers of *AFP* or *APOA4*, then upregulated mature hepatocyte transcription factors, such as *PROX1*, *HHEX*, and nuclear receptors such as *NR1H4* (encodes the bile acid receptor *FXR*), and *NR1I3* (encodes the *constitutive androstane receptor*, *CAR)*. Upregulation of nuclear receptors is important in that many target genes important for hepatocytic differentiation and maturation are regulated by nuclear receptors. *NR1H4* is an important transcription factor that regulates bile acid synthesis, detoxification and transport, and is also involved in the transcriptional regulation of *CYP3A4*, *CYP7A1*, *SLCO1B1* (Calkin and Tontonoz, 2012). *NR1I3* influences the expression of a broad spectrum of the metabolizing phase I *CYP* enzymes, and enzymes involved in phase II drug metabolism, as well as the transporter proteins (Kandel et al., 2016). These features led to the upregulation of mature hepatocyte markers *ALB*, *ASGR1* and *SERPINA1* at later stages of hiPS cell differentiation compared to those differentiated on nanofiber matrices. The drug uptake transporters *SLCO1B1*, *SLC22A1*, phase II enzymes *UGT1A1*, excretion transporter *ABCC3* and many *CYP* enzymes are expressed in the hiPS-hep. CYP enzymes such as *CYP1A1*, *CYP1A2*, *CYP2A6*, *CYP2C8*, *CYP2D6*, *CYP2E1* and *CYP3A4* that are involved in the metabolism of a significant proportion of the drugs in the liver (Terada and Hira, 2015), are expressed at a substantial level in the hiPS-hep grown on the CV membrane. The hiPS-hep expressed *CYP3A4* at about 1/50-fold of that of PH (no pre-culture). Particularly important is that the metabolic enzyme activities of CYP1A, CYP2D6, CYP2C9, CYP2C19 and CYP3A were observed, among which, CYP1A and CYP3A activity in hiPS-hep were higher than that of PH (pre-4 h), CYP2C19 activity was higher than the PH pre-48 h, and CYP2D6 activity was equivalent to that of PH pre-48 h. Enzyme activities were observed from D32 up to D47 hiPS-hep, thereby suggesting that the metabolic enzyme activities were maintained for at least 2 weeks. Notably, CYP3A4, CYP3A5 and CYP3A7 gene expressions also resemble those of the endogenous hepatocytes.

We also examined the gene expressions of the zone-specific markers (Kietzmann, 2017; Si-Tayeb et al., 2010b). Expression levels of hepatocyte zone 1 markers, such as *CPS1* or a gluconeogenesis-related gene *PCK2* were not upregulated, whereas *G6PC* (Fig. S3) and urea production related genes *ASL1* and *ARG1* were upregulated (Fig. 4). As noted above, hepatocyte zone 3 markers such as the CYP enzymes (Fig. 3) and *AHR* (Fig. S3) were upregulated. The expression levels of some of the hepatocyte zone 1 markers are not upregulated. This feather might attribute to a lack of bile duct formation in our present culture condition. Recent reports describe the segregation of human liver bud progenitors into hepatocyte versus biliary fates, in which the signaling events involved might be useful for future studies for the generation of a full range of different functional hepatocytes (Ang et al., 2018; Sampaziotis et al., 2017).

We previously reported the importance of basement membrane substratum in potentiation of differentiation of hES/hiPS cells through activation of integrin beta 1. hES/hiPS cells sense and transmit extracellular signals, and secrete heparin sulfate proteoglycans (HSPGs), which acts at the vicinity of the cells with growth factor receptors to mediate inducing signals (Higuchi et al., 2010; Shiraki et al., 2011). It was previously reported that dedifferentiation of hepatocytes and a reduction of liver functions in a 2D cell culture, and that cells in the liver are mechanosensitive, and the stiffness of the matrix is important (Godoy et al., 2009; Wells, 2008). The matrix that provides a 3D microenvironment mimicking the extracellular matrices could support hES/hiPS cell differentiation (Gieseck et al., 2014; Yamazoe et al., 2013). CV membrane was reported to be useful to induce HepG2 cells to form the hepatic structure that exhibited CYP3A4 activity and supported long term culture of hepatocytes (Oshikata-Miyazaki and Takezawa, 2016; Watari et al., 2018), which might be an important key for its potentiation for maturation of the hiPS-hep. We also observed enhanced differentiation into hepatic lineage on the CV membrane upon replating of the hiPS-endo, compared to those plated on collagen I transwells (S.N., unpublished). Taken together, the CV membrane provides a suitable environment mimicking *in vivo* and potentiated differentiation and maturation of the hiPS-hep.

Another point to be noted in our current protocol is the use of frozen hiPS-endo cryopreserved cells. M15 was shown to derive endoderm cells from hES/hiPS cells efficiently (Shiraki et al., 2008a). By using the M15 system, a large number of hiPS-endo could be obtained efficiently, which allowed rapid differentiation of the hiPS-hep. Generally, we could obtain 4×10^7^ hiPS-endo from one batch of M15 feeders on a 100 mm diameter plate, starting from 5×10^5^ undifferentiated hiPS cells.

In this study, we established an efficient procedure to differentiate hiPS cells into mature hepatocytes by culturing on collagen vitrigel membrane. The derived hiPS-hep exhibited a high metabolic CYP enzyme activity of CYP2C19 and CYP3A, which are comparable to that of PH. Our results thereby suggesting the hiPS-hep generated resemble that of the hepatocytes and provide an excellent model for *in vitro* liver toxicity studies as well as disease modeling.

## MATERIALS AND METHODS

### Ethical approval

Since the primary human hepatocytes or hiPS cells used were purchased from vendors, there was no need to go through institutional ethical committee involving human materials.

### hiPS cell lines

The hiPS cell line, ChiPS18 (Asplund et al., 2015) (TakaraBio, Kyoto) was used. Undifferentiated hiPS cells were maintained in AK02 StemFit media (Ajinomoto) on cell culture dishes (Invitrogen) pre-coated with Synthemax II (Corning, cat no. 3535). For methionine deprivation, hiPS cells were cultured in StemFit media (Ajinomoto) complete (as a control), or Met-deprived KA01 medium (Ajinomoto).

### Preparation of collagen vitrigel membrane chambers

A collagen xerogel membrane is manufactured by Kanto Chemical Co., Inc. (Tokyo, Japan). Briefly, collagen vitrigel membrane was prepared by the following three processes as previously reported (Oshikata-Miyazaki and Takezawa, 2016; Yamaguchi et al., 2013, 2016): (1) a gelation process in which 2.0 ml of 0.25% type I collagen sol was allowed to form an opaque soft gel in a culture dish with a diameter of 35 mm; (2) a vitrification process in which the gel became a rigid material through sufficient drying; and (3) a rehydration process in which the vitrified material was converted into a thin transparent gel membrane with enhanced gel strength by moisture supplementation. Subsequently, a collagen xerogel membrane defined as a dried collagen vitrigel membrane without free water was prepared by simply revitrifying a collagen vitrigel membrane on a separable sheet. The collagen xerogel membrane was pasted onto the bottom edge of a plastic cylinder with an inner–outer diameter of 11–13 mm and a length of 15 mm, and two hangers were connected to the top edge of the cylinder, resulting in the fabrication of a collagen xerogel membrane chamber that easily allowed conversion into a collagen vitrigel membrane chamber by rehydration with culture media, as previously reported (Oshikata-Miyazaki and Takezawa, 2016; Yamaguchi et al., 2013, 2016).

### Differentiation of hiPS cells into hepatocytic lineages

Undifferentiated hiPS cells were first differentiated into the endoderm using M15 cells.

Briefly, 100 mm diameter plates were pre-coated with mitomycin-treated frozen M15 feeder cells at a density of 5×10^6^ cells/dish. For endoderm differentiation, undifferentiated ChiPS18 cells were plated onto M15 cell-coated 100 mm diameter plates at a density of 5×10^5^ cells/dish and cultured in methionine deprived medium StemFit KA01 medium for 5 h (Ajinomoto), then switched into endoderm differentiation medium M1 supplemented with 3 µM CHIR99021 (Wako, Tokyo) for 1 day, then changed to M1 without CHIR99021 and cultured for another 2 days. M1 consists of DMEM, 4500 mg/l glucose, non-essential amino acids (NEAA, Thermo Fisher), L-Gln, 1% Penicillin, Streptomycin solution (PS; Nacalai Tesque), 0.1 mM β-ME (Sigma), serum-free B27 supplement (#12587001, Thermo Fisher), 100 ng/ml recombinant human activin A (Cell Guidance Systems, Cambridge, UK). On day 3 (D3), ChiPS18-derived endodermal cells were collected and frozen at 2.0×10^6^ cells/ml in Bambanker hRM (NIPPON Genetics Co, Ltd CS-07-001) and stocked in liquid N_2_ until further use. All cell numbers noted throughout the text are viable cells.

For hepatocytic differentiation, frozen endodermal cells were freeze-thawed and plated onto rehydrated vitrigel (CV) membrane 24-well inserts (ad-MED Vitrigel™ 2, Kanto Chemical Co., Inc., culture area: 0.33 cm^2^/insert), at a concentration of 1×10^5^ cells/well. The volume of the media was 200 μl for the upper layer and 500 μl for the lower layer of the transwells. Media in both upper and lower layers were replaced every 2 days with fresh medium and growth factors. Media used for differentiation were: M2 for D3–D7 was changed to M3 for D7–D15, then changed to M4 media, for D15∼D30 or up to D40. M2 consists of knockout DMEM/F12 (Thermo Fisher), supplemented with NEAA, L-Gln, PS, 0.1 mM β-ME, serum-free B27 supplement, 10 ng/ml recombinant human FGF10 and 10 ng/ml recombinant human BMP4. M3 consists of HCM SingleQuot Kit™ (Lonza CC-4182) supplemented with 50 ng/ml human recombinant HGF (Pepro Tech), and 20 ng/ml Oncostatin M (Sigma). For M4 media, Cellartis^®^ Hepatocyte Maintenance Medium (MM medium, Takara Bio Y30051) was used.

For differentiation on 2D plates or nanofiber plates, day 3 DE were replated onto 24 well Corning CellBIND plates (Corning) pre-coated with Synthemax II (Corning) or 24-well nanofiber plates pre-coated with Matrigel (BD) (Yamazoe et al., 2013), respectively.

### Immunocytochemistry

Cells were fixed in 4% paraformaldehyde (Nacalai Tesque) in PBS, permeabilized with 0.1% Triton X-100 (Nacalai Tesque), and were then blocked with 20% Blocking One (Nacalai Tesque, Japan) in PBST (0.1% Tween-20 in PBS). Antibodies were diluted in 20% Blocking One (Nacalai Tesque, Japan) in PBST (0.1% Tween-20 in PBS). Cells were counterstained with 6-diamidino-2-phenylindole (DAPI) (Roche Diagnostics, Switzerland).

The following antibodies were used: rabbit anti-AFP (Dako, Glostrup, Denmark), goat anti-ALB (Bethyl), mouse anti-OCT3/4 (Santa Cruz), goat anti-SOX17 (R&D Systems), and Alexa 568-conjugated and Alexa 488-conjugated antibodies (Invitrogen). Positive cells versus total cells (DAPI-positive cells) were quantified using the Metamorph Image Analysis Software (Molecular Devices).

### RT-PCR analysis

RNA was extracted from hiPS cells using the RNeasy micro-kit or Qiaxol (Qiagen, Germany) and then treated with DNase (Qiagen). For reverse transcription reactions, 2.5 µg RNA was reverse-transcribed using PrimeScript™ RT Master Mix (Takara, Japan). For real-time PCR analysis, the mRNA expression was quantified with SyberGreen on a StepOne Plus (Applied Biosystems, Foster City, USA). The PCR conditions were as follows: initial denaturation at 95°C for 30 s, followed by denaturation at 95°C for 5 s, annealing and extension at 60°C for 30 s, for up to 40 cycles. Target mRNA levels were expressed as arbitrary units and were determined using the ΔΔ CT method. PrimerArray Hepatic Differentiation (Human) (TaKaRa PH017) containing primer pairs optimized for real-time RT-PCR analysis of 88 genes associated with hepatic differentiation and eight housekeeping genes was used. Primer details are listed in Table S1.

### Principal component analysis of the primer array data

PCA was performed to visualize the results and map the expression profile of the primer array results and microarray results. PCA was performed using JMP software (SAS Institute Japan).

### Microarray analysis

Undifferentiated hiPS cells (D0), hiPS-endo (D3), hiPS-hepatoblast (D15) and hiPS-hep (D30), as well as cryopreserved primary hepatocytes, were harvested and total RNA extracted was checked using a 2100 Bioanalyzer. Samples that passed a quality check were used for microarray analysis. Microarray analysis was performed by the Chemicals Evaluation and Research Institute, Japan (CERI). Briefly as followings: SurePrint G3 Human GE microarray 8x60K Ver.2.0 (Agilent) was used, performed under the protocol of One-Color Microarray-Based Gene Expression Analysis (Low Input Quick Amp Labeling), ver6.9, December 2015 (Agilent). The analysis was done with GeneSpring GX14.5 (Agilent).

Principal Component Analysis using GeneSpring GX14.5 (Agilent) and IPA Version 42012434 (QIAGEN) were performed. Hierarchical clustering analysis through Ward's minimum variance linkage on normalized expression data was performed.

### Human primary hepatocytes

Commercially available cryopreserved primary hepatocytes (PH) were used (BioreclamationIVT, Cat. No. M00995-P, Lot no. FOS, >5.0×10^6^ cells/vial). For real-time PCR analysis using PrimerArray Hepatic Differentiation, RNA was extracted from cryopreserved primary hepatocytes without pre-culture. For CYP activity, cryopreserved primary hepatocytes were thawed and resuspended at a concentration of 7.0×10^5^ living cells/ml, in InVitroGRO™ CP Medium mixed with Torpedo™ Antibiotic Mix, following manufacturer's instruction (BioreclamationIVT), and were plated onto BioCoat™ collagen I 24-well microplates (Corning), at 3.5×10^5^ cells/well. Media was replaced with William's E medium containing CM4000 (without phenol red; Life Technologies), after 4∼6 h, and on the next day. CYP activity assays were started 4 h (pre-4 h) or 48 h (pre-48 h) after plating.

### CYP activity assay

CYP1A, CYP2C9, CYP2C19, CYP2D6 and CYP3A activities in hiPS-hep on day 32 and day 47 of differentiation, were evaluated. PHs plated for 4 h or 48 h were used as references. hiPS-hep or PHs cultures were washed with warm William's E media supplemented with PS. Assays were started by adding media containing a cocktail of mixture at final concentrations of the following chemicals: 50 µM phenacetin (Sigma), 5 µM diclofenac Na (Wako), 100 µM *S*-mephenytoin (Sumika Chemical Analysis Service, Ltd), 10 µM bufuralol HCl (Sumika Chemical Analysis Service, Ltd) and 5 µM midazolam (Wako). The volume of media added was 500 µl to the PH, and 300 µl to the upper and 600 µl to the lower chamber of the transwell CV membrane. After 1, 2, 6 and 24 h, 100 µl or 300 µl of supernatants were collected from both upper and lower chamber, respectively, and kept at −80°C, until performing LC/MS/MS analysis of the metabolites, acetaminophen, 1′-OH bufuralol, 4′-OH diclofenac, 4-OH mephenytoin, and 1-OH midazolam. For hiPS-hep, the chemicals were added into both the upper and lower chambers, and supernatants were collected and combined from both chambers. Protein amount per well was quantified using the Pierce BCA protein assay kit (Thermo Fisher) according to the manufacturer's instructions. Metabolite concentrations were normalized with protein amounts. hiPS cell differentiation, PHs culture, the addition of chemicals and sampling of the reaction mixture, except LC/MS/MS, were done in Tokyo Institute of Technology, and then sent to different institutions for LC/MS/MS analysis of the metabolites.

### Bioanalysis by liquid chromatography and tandem mass spectrometry

For detection by Chugai Pharmaceutical Corporate, Ltd, the collected incubating media were extracted by adding acetonitrile/isopropanol (1/1, v/v) containing an internal standard, and were analyzed using AQUITY UPLC (Waters Corp.) and XevoTQ-S (Waters Corp.) and YMC-Triart column (30×2.0 mm I.D., YMC Co., Ltd).

For detection by Taisho Pharmaceutical Co., Ltd, the collected incubating media were mixed with 0.1% formic acid containing acetonitrile with the internal standard and analyzed using a system consisting of a TripleQuad 5500 (AB Sciex, Foster City, USA) and an HPLC system consisting of two LC-30AD series HPLC pumps, a SIL-30AC autosampler, a CTO- 20AC column oven (Shimadzu, Kyoto, Japan) and a Shim-pack XR-ODS 3.0×30 mm, 2.2-µm column (Shimadzu, Kyoto, Japan).

For detection by Toray Industries, Inc., the collected incubating media were extracted by adding acetonitrile containing an internal standard, and were quantified by LC/MS/MS with the liquid chromatography, ACQUITY UPLC I-class system (Waters Corp.) and the mass spectrometer, API-5000 (SCIEX), using CAPCELLPAK C18 MGIII, 50×2.0 mm, 5 µm column (Shiseido Co. Ltd.).

### Statistics

Data are expressed as the mean±s.d. (*n*=3). Differences between groups were analyzed by Student's *t*-tests or one-way ANOVA with the post-hoc Dunnett's test. The respective statistical analysis and *P*-value are noted in each figure legend. *P<0.05 or **P<0.01, by Student's *t*-test and ^§^*P*<0.05 or ^§§^*P*<0.01, by one-way ANOVA with the post-hoc Dunnett's test are considered to be significant.

## Supplementary Material

Supplementary information
