# Supplementary material for: Collagen vitrigel promotes hepatocytic differentiation of induced pluripotent stem cells into functional hepatocyte-like cells
Source: Biol Open. 2019 Jun 10;8(7):bio042192. doi: 10.1242/bio.042192 (PMC6679405; doi:10.1242/bio.042192)
Supplement: Supplementary information [file biolopen-8-042192-s1.pdf]

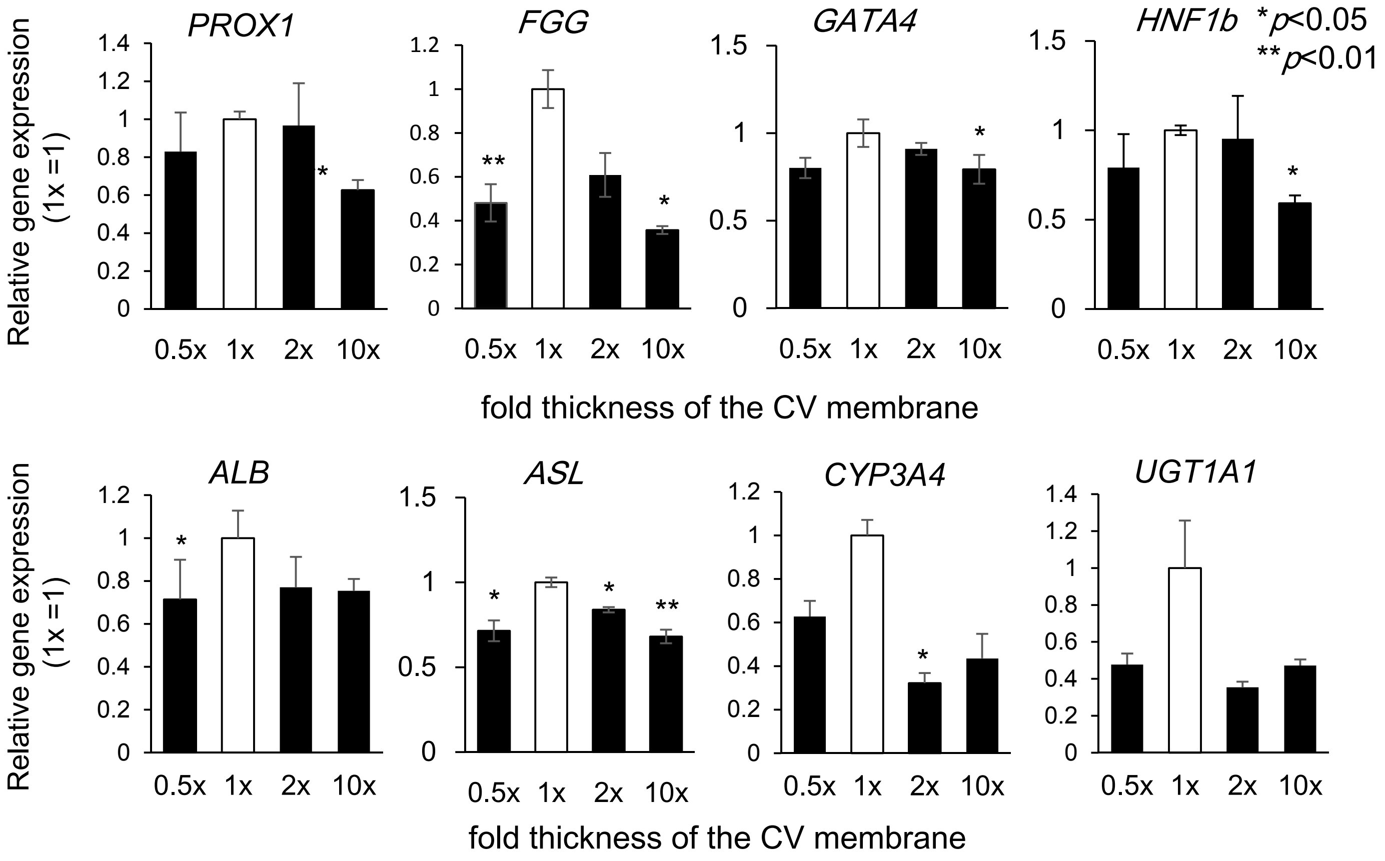

Figure S1. Comparison among different thicknesses of the CV membrane used

Human iPS cell-derived endoderm cells on day 3 were plated onto 0.5x, 1x, 2x, and 10x-fold thicknesses of CV membrane and cultured until day 32. Expression levels of various gene transcripts are quantified by real-time RT-PCR in the differentiated cells. Values represent means ± S. D (n=3). Significance shown as $^{*}p<0.05$ or $^{**}p<0.01$, by two tailed Student's *t*-test, compared to those of hiPS-hep grown on 1x thickness of CV.

Biology Open • Supplementary information

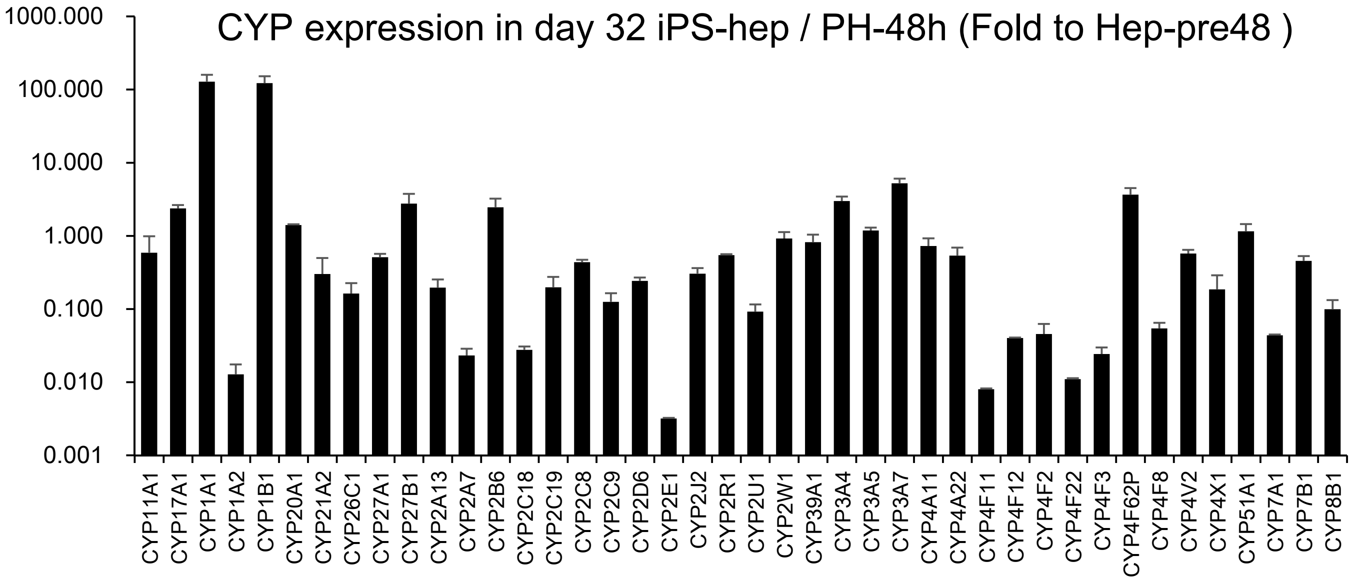

Figure S2. *CYP* expression profiles revealed by gene array analysis of hiPS-hep on day 30 versus PH (pre-48h).

CYP genes expressed in day 30 hiPS-hep are shown as fold changes to those expressed in PH (pre-48h). Values represent means ± S. D (n=3).

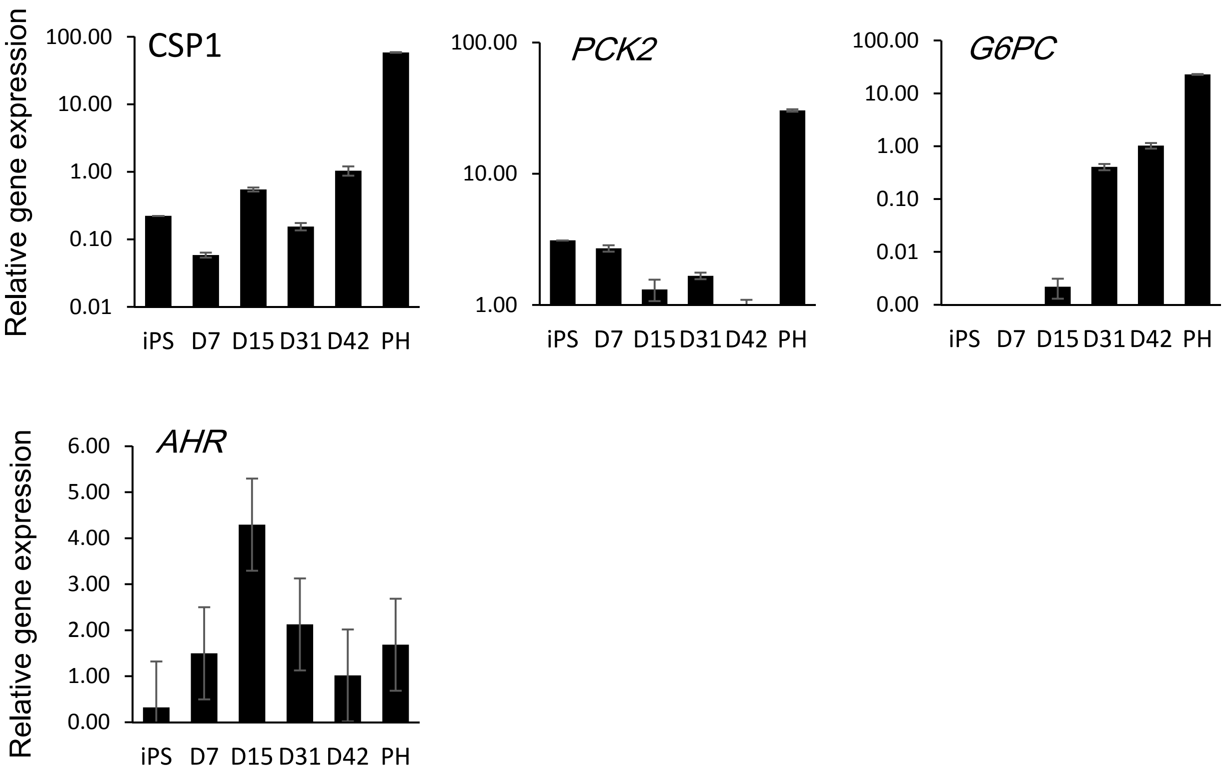

Figure S3. Expression levels of the hepatocyte zone 1 (CPS1, PCK2, G6PC) and 3 (AHR) genes.

Expression levels of various gene transcripts quantified by real-time RT-PCR in undifferentiated hiPS cells (iPS) or D7, D15, D31 or D42 differentiated hiPS cells on the CV membrane are compared with that of the primary hepatocytes (PH, 0h; no pre-culture). Values represent means ± S. D (n=3).

Biology Open • Supplementary information

Biology Open (2019): doi:10.1242/bio.042192: Supplementary information

Table S1. Primers list used in PrimerArray for detecting human hepatic differentiation

Well ID	Symbol	Genbank Acc	Description	Gene size	Position	Oligo dT	Random 6 mers	Exon_junction	Variants	Variants cover ratio
1	NANOG	NM_024865.2	Homo sapiens Nanog homeobox (NANOG), mRNA.	2098	375	NG	OK	1/3		1/1
2	POU5F1	NM_002701.4	Homo sapiens POU class 5 homeobox 1 (POU5F1), transcript variant 1, mRNA.	1411	508	OK	OK	2/4		1/3
3	SOX2	NM_003106.3	Homo sapiens SRY (sex determining region Y)-box 2 (SOX2), mRNA.	2520	1081	OK	OK	0/0		1/1
4	CD9	NM_001769	Homo sapiens CD9 molecule (CD9), mRNA.	1321	238	OK	OK	1/7		1/1
5	DNMT3B	NM_175848	Homo sapiens DNA (cytosine-5-)-methyltransferase 3 beta (DNMT3B), transcript variant 2, mRNA.	4293	1257	NG	OK	1/21	NM_006892,NM_175849,NM_175850,NM_001207055,NM_001207056	6/6
6	GABRB3	NM_000814	Homo sapiens gamma-aminobutyric acid (GABA) A receptor, beta 3 (GABRB3), transcript variant 1, mRNA.	5783	677	NG	OK	1/8	NM_021912,NM_001191320,NM_001191321	4/4
7	GAL	NM_015973	Homo sapiens galanin prepropeptide (GAL), mRNA.	778	350	OK	OK	1/5		1/1
8	GDF3	NM_020634	Homo sapiens growth differentiation factor 3 (GDF3), mRNA.	1224	337	OK	OK	1/1		1/1
9	GRB7	NM_005310	Homo sapiens growth factor receptor-bound protein 7 (GRB7), transcript variant 1, mRNA.	2285	1112	OK	OK	1/14	NM_001030002,NM_001242442,NM_001242443	4/4
10	IFITM1	NM_003641	Homo sapiens interferon induced transmembrane protein 1 (9-27) (IFITM1), mRNA.	733	423	OK	OK	1/1		1/1
11	PODXL	NM_005397	Homo sapiens podocalyxin-like (PODXL), transcript variant 2, mRNA.	5911	1269	NG	OK	1/7	NM_001018111	2/2
12	TDGF1	NM_001174136	Homo sapiens teratocarcinoma-derived growth factor 1 (TDGF1), transcript variant 2, mRNA.	1784	428	OK	OK	1/5	NM_003212	2/2
13	ZFP42	NM_174900	Homo sapiens zinc finger protein 42 homolog (mouse) (ZFP42), mRNA.	2646	308	NG	OK	1/3		1/1
14	SOX7	NM_031439.2	Homo sapiens SRY (sex determining region Y)-box 7 (SOX7), mRNA.	3219	280	NG	OK	1/1		1/1
15	LAMB1	NM_002291.2	Homo sapiens laminin, beta 1 (LAMB1), mRNA.	5866	4733	OK	OK	1/33		1/1
16	HNF1B	NM_000458.2	Homo sapiens HNF1 homeobox B (HNF1B), transcript variant 1, mRNA.	2842	1787	OK	OK	1/8	NM_001165923	2/3
17	GSC	NM_173849.2	Homo sapiens goosecoid homeobox (GSC), mRNA.	1217	800	OK	OK	1/2		1/1
18	NODAL	NM_018055.4	Homo sapiens nodal homolog (mouse) (NODAL), mRNA.	2086	946	OK	OK	1/2		1/1
19	FOXA2	NM_021784.4	Homo sapiens forkhead box A2 (FOXA2), transcript variant 1, mRNA.	2428	286	NG	OK	1/1	NM_153675	2/2
20	SOX17	NM_022454.3	Homo sapiens SRY (sex determining region Y)-box 17 (SOX17), mRNA.	2350	1548	OK	OK	0/1		1/1
21	CXCR4	NM_001008540.	Homo sapiens chemokine (C-X-C motif) receptor 4 (CXCR4), transcript variant 1, mRNA.	1912	992	OK	OK	0/0	NM_003467	2/2
22	GATA4	NM_002052.3	Homo sapiens GATA binding protein 4 (GATA4), mRNA.	3419	1542	NG	OK	1/6		1/1
23	GATA6	NM_005257.4	Homo sapiens GATA binding protein 6 (GATA6), mRNA.	3770	1890	NG	OK	1/6		1/1
24	AFP	NM_001134.1	Homo sapiens alpha-fetoprotein (AFP), mRNA.	2032	332	NG	OK	1/14		1/1
25	CYP3A7	NM_000765	Homo sapiens cytochrome P450, family 3, subfamily A, polypeptide 7	2095	987	OK	OK	2/12		1/1
26	DLK1	NM_003836.5	Homo sapiens delta-like 1 homolog (Drosophila) (DLK1), mRNA.	1599	316	OK	OK	2/4		1/2
27	PROX1	NM_002763.3	Homo sapiens prospero homeobox 1 (PROX1), mRNA.	3088	2265	OK	OK	1/4		1/1
28	TBX3	NM_005996.3	Homo sapiens T-box 3 (TBX3), transcript variant 1, mRNA.	4754	1887	NG	OK	1/6	NM_016569	2/2
29	SERPINA1	NM_000295.4	Homo sapiens serpin peptidase inhibitor, clade A (alpha-1 antiproteinase, antitrypsin), member 1 (SERPINA1), transcript variant 1, mRNA.	3220	1850	OK	OK	0/4	NM_001002235,NM_001002236,NM_001127700,NM_001127701,NM_001127702, NM_001127703,NM_001127704,NM_001127705,NM_001127706,NM_001127707	11/11
30	AHSG	NM_001622.2	Homo sapiens alpha-2-HS-glycoprotein (AHSG), mRNA.	1594	269	OK	OK	1/6		1/1
31	ALB	NM_000477.5	Homo sapiens albumin (ALB), mRNA.	2264	310	NG	OK	1/14		1/1
32	APOA4	NM_000482.3	Homo sapiens apolipoprotein A-IV (APOA4), mRNA.	1460	258	OK	OK	1/2		1/1
33	ASGR1	NM_001671.4	Homo sapiens asialoglycoprotein receptor 1 (ASGR1), transcript variant 1, mRNA.	1519	394	OK	OK	1/8	NM_001197216	2/2
34	KRT8	NM_001256282.	Homo sapiens keratin 8 (KRT8), transcript variant 1, mRNA.	1807	73	NG	OK	1/8		1/4
35	KRT18	NM_000224.2	Homo sapiens keratin 18 (KRT18), transcript variant 1, mRNA.	1485	1259	OK	OK	1/6	NM_199187	2/2
36	FABP1	NM_001443.2	Homo sapiens fatty acid binding protein 1, liver (FABP1), mRNA.	598	441	OK	OK	1/3		1/1
37	FGG	NM_000509.4	Homo sapiens fibrinogen gamma chain (FGG), transcript variant gamma-A, mRNA.	1665	1275	OK	OK	1/9	NM_021870	2/2
38	G6PC	NM_000151.2	Homo sapiens glucose-6-phosphatase, catalytic subunit (G6PC), mRNA.	3096	320	NG	OK	1/4		1/1
39	PCK2	NM_004563.2	Homo sapiens phosphoenolpyruvate carboxykinase 2 (mitochondrial) (PCK2), nuclear gene encoding mitochondrial protein, transcript variant 1,	2221	413	NG	OK	1/9	NM_001018073	2/2
40	RBP4	NM_006744.3	Homo sapiens retinol binding protein 4, plasma (RBP4), mRNA.	941	442	OK	OK	1/5		1/1
41	TAT	NM_000353.2	Homo sapiens tyrosine aminotransferase (TAT), nuclear gene encoding mitochondrial protein, mRNA.	2757	1381	OK	OK	1/11		1/1
42	TF	NM_001063.3	Homo sapiens transferrin (TF), mRNA.	2808	2009	OK	OK	1/16		1/1
43	TDO2	NM_005651.3	Homo sapiens tryptophan 2,3-dioxygenase (TDO2), mRNA.	1703	494	OK	OK	1/11		1/1
44	HHEX	NM_002729.4	Homo sapiens hematopoietically expressed homeobox (HHEX), mRNA.	1772	675	OK	OK	1/3		1/1
45	HNF1A	NM_000545.5	Homo sapiens HNF1 homeobox A (HNF1A), mRNA.	3241	2917	OK	OK	0/9		1/1
46	HNF4A	NM_178849.2	Homo sapiens hepatocyte nuclear factor 4, alpha (HNF4A), transcript variant 1, mRNA.	4707	905	NG	OK	1/9	NM_001030003,NM_001030004,NM_175914,NM_000457,NM_178850,NM_001258355	7/7
47	AHR	NM_001621.4	Homo sapiens aryl hydrocarbon receptor (AHR), mRNA.	6247	1479	NG	OK	1/10		1/1
48	NR1I3	NM_001077473.	Homo sapiens nuclear receptor subfamily 1, group I, member 3 (NR1I3), transcript variant 12, mRNA.	1120	607	OK	OK	1/7	NM_001077480,NM_001077481,NM_001077482,NM_001077474,NM_001077475,NM_001077476,NM_001077477, NM_001077478,NM_001077479,NM_001077469,NM_005122,NM_001077470,NM_001077471,NM_001077472	15/15
49	NR1I2	NM_003889.3	Homo sapiens nuclear receptor subfamily 1, group I, member 2 (NR1I2), transcript variant 1, mRNA.	4446	2660	NG	OK	1/8	NM_033013,NM_022002	3/3
50	NR1H3	NM_005693.3	Homo sapiens nuclear receptor subfamily 1, group H, member 3 (NR1H3), transcript variant 1, mRNA.	1939	1250	OK	OK	1/9	NM_001130101,NM_001130102,NM_001251934,NM_001251935	5/5
51	NR1H4	NM_001206979.	Homo sapiens nuclear receptor subfamily 1, group H, member 4 (NR1H4), transcript variant 1, mRNA.	2325	1412	OK	OK	1/10	NM_001206977,NM_001206978,NM_005123,NM_001206993,NM_001206992	6/6
52	RXRA	NM_002957.4	Homo sapiens retinoid X receptor, alpha (RXRA), mRNA.	5547	180	NG	OK	1/9		1/1
53	NR3C1	NM_001204259.	Homo sapiens nuclear receptor subfamily 3, group C, member 1 (glucocorticoid receptor) (NR3C1), transcript variant 1, mRNA.	6801	2026	NG	OK	1/8	NM_001024094,NM_001204258,NM_001204260,NM_001204261,NM_001204262,NM_001204263,NM_001204264, NM_001204265,NM_000176,NM_001018074,NM_001018075,NM_001018076,NM_001018077,NM_001020825	15/15
54	PPARA	NM_001001928.	Homo sapiens peroxisome proliferator-activated receptor alpha (PPARA), transcript variant 3, mRNA.	9965	552	NG	OK	1/7	NM_005036	2/5
55	CYP1A1	NM_000499	Homo sapiens cytochrome P450, family 1, subfamily A, polypeptide 1	2608	1971	OK	OK	0/6		1/1
56	CYP1A2	NM_000761	Homo sapiens cytochrome P450, family 1, subfamily A, polypeptide 2	3127	969	NG	OK	0/6		1/1
57	CYP2A6	NM_000762	Homo sapiens cytochrome P450, family 2, subfamily A, polypeptide 6 (CYP2A6), mRNA	1775	1212	OK	OK	1/8		1/1
58	CYP2C8	NM_000770	Homo sapiens cytochrome P450, family 2, subfamily C, polypeptide 8, transcript variant 1	1924	1389	OK	OK	1/8	NM_001198853,NM_001198854,NM_001198855	4/5
59	CYP2E1	NM_000773	Homo sapiens cytochrome P450, family 2, subfamily E, polypeptide 1	1667	1026	OK	OK	1/8		1/1
60	CYP2D6	NM_000106	Homo sapiens cytochrome P450, family 2, subfamily D, polypeptide 6 ,	1673	1419	OK	OK	1/8	NM_001025161	2/2
61	CYP3A4	NM_017460	Homo sapiens cytochrome P450, family 3, subfamily A, polypeptide 4	2792	1595	OK	OK	1/12	NM_001202855	2/4
62	CYP7A1	NM_000780	Homo sapiens cytochrome P450, family 7, subfamily A, polypeptide 1	2875	235	NG	OK	1/5		1/1
63	ABCB4	NM_000443.3	Homo sapiens ATP-binding cassette, sub-family B (MDR/TAP), member 4 (ABCB4), transcript variant A, mRNA.	3967	2627	OK	OK	1/27	NM_018850,NM_018849	3/3
64	ABCC1	NM_004996.3	Homo sapiens ATP-binding cassette, sub-family C (CFTR/MRP), member 1 (ABCC1), mRNA.	6564	1681	NG	OK	1/30		1/7
65	ABCC3	NM_003786.3	Homo sapiens ATP-binding cassette, sub-family C (CFTR/MRP), member 3 (ABCC3), transcript variant 1, mRNA.	5183	1530	NG	OK	1/30	NM_001144070	2/4
66	ABCG2	NM_004827.2	Homo sapiens ATP-binding cassette, sub-family G (WHITE), member 2 (ABCG2), transcript variant 1, mRNA.	4445	1394	NG	OK	2/15	NM_001257386	2/2
67	SLC10A1	NM_003049.3	Homo sapiens solute carrier family 10 (sodium/bile acid cotransporter family), member 1 (SLC10A1), mRNA.	1631	524	OK	OK	1/4		1/1
68	SLC22A1	NM_003057.2	Homo sapiens solute carrier family 22 (organic cation transporter), member 1 (SLC22A1), transcript variant 1, mRNA.	1921	790	OK	OK	1/10	NM_153187	2/2
69	SLC22A2	NM_003058.3	Homo sapiens solute carrier family 22 (organic cation transporter), member 2 (SLC22A2), mRNA.	2512	1464	OK	OK	1/10		1/2
70	SLCO1B1	NM_006446.4	Homo sapiens solute carrier organic anion transporter family, member 1B1 (SLCO1B1), mRNA.	2800	1577	OK	OK	1/14		1/1
71	SLCO1B3	NM_019844.3	Homo sapiens solute carrier organic anion transporter family, member 1B3 (SLCO1B3), mRNA.	3014	2007	OK	OK	1/15		1/1
72	SLCO2B1	NM_007256.4	Homo sapiens solute carrier organic anion transporter family, member 2B1 (SLCO2B1), transcript variant 1, mRNA.	4362	3036	OK	OK	0/13	NM_001145211,NM_001145212	3/3
73	UGT1A1	NM_000463.2	Homo sapiens UDP glucuronosyltransferase 1 family, polypeptide A1 (UGT1A1), mRNA.	2357	430	NG	OK	0/4		1/1
74	GSTA2	NM_000846.4	Homo sapiens glutathione S-transferase alpha 2 (GSTA2), mRNA.	1320	432	OK	OK	1/6		1/1
75	ATP5G1	NM_005175.2	Homo sapiens ATP synthase, H+ transporting, mitochondrial Fo complex, subunit C1 (subunit 9) (ATP5G1), nuclear gene encoding mitochondrial protein, transcript variant 1, mRNA.	663	455	OK	OK	1/4	NM_001002027	2/2
76	POLG	NM_002693.2	Homo sapiens polymerase (DNA directed), gamma (POLG), transcript variant 1, mRNA.	4464	3732	OK	OK	1/22	NM_001126131	2/2
77	PPARGC1A	NM_013261.3	peroxisome proliferator-activated receptor gamma, coactivator 1 alpha (PPARGC1A)	6318	521	NG	OK	1/12		1/1
78	UCP2	NM_003355.2	Homo sapiens uncoupling protein 2 (mitochondrial, proton carrier) (UCP2), nuclear gene encoding mitochondrial protein, mRNA.	1646	1209	OK	OK	1/7		1/1
79	ARG1	NM_001244438.	Homo sapiens arginase, liver (ARG1), transcript variant 1, mRNA.	1499	663	OK	OK	1/7	NM_000045	2/2
80	ASL	NM_001024943.	Homo sapiens argininosuccinate lyase (ASL), transcript variant 1, mRNA.	2061	1295	OK	OK	1/15	NM_000048,NM_001024944,NM_001024946	4/4
81	ASS1	NM_000050.4	Homo sapiens argininosuccinate synthase 1 (ASS1), transcript variant 1, mRNA.	1863	1356	OK	OK	1/15	NM_054012	2/2
82	CPS1	NM_001122633.	Homo sapiens carbamoyl-phosphate synthase 1, mitochondrial (CPS1), nuclear gene encoding mitochondrial protein, transcript variant 1, mRNA.	5738	4340	OK	OK	1/38	NM_001875,NM_001122634	3/3
83	NAGS	NM_153006.2	Homo sapiens N-acetylglutamate synthase (NAGS), mRNA.	2086	1459	OK	OK	1/6		1/1
84	OTC	NM_000531.5	Homo sapiens ornithine carbamoyltransferase (OTC), nuclear gene encoding mitochondrial protein, mRNA.	1647	67	NG	OK	0/9		1/1
85	KRT19	NM_002276.4	Homo sapiens keratin 19 (KRT19), mRNA.	1490	840	OK	OK	1/5		1/1
86	KRT7	NM_005556.3	Homo sapiens keratin 7 (KRT7), mRNA.	1753	1008	OK	OK	1/8		1/1
87	AQP1	NM_198098.2	Homo sapiens aquaporin 1 (Colton blood group) (AQP1), transcript variant 1, mRNA.	2807	775	NG	OK	1/3	NM_001185060,NM_001185061,NM_001185062	4/5
88	HES1	NM_005524.3	Homo sapiens hairy and enhancer of split 1, (Drosophila) (HES1), mRNA.	1475	516	OK	OK	1/3		1/1
89	GUSB	NM_000181	Homo sapiens glucuronidase, beta (GUSB), mRNA.	2321	1773	OK	OK	1/11		1/1
90	HPRT1	NM_000194	Homo sapiens hypoxanthine phosphoribosyltransferase 1 (HPRT1), mRNA.	1435	684	OK	OK	2/8		1/1
91	PGK1	NM_000291	Homo sapiens phosphoglycerate kinase 1 (PGK1), mRNA.	2439	1086	OK	OK	1/10		1/1
92	ACTB	NM_001101.3	Homo sapiens actin, beta (ACTB), mRNA.	1852	1135	OK	OK	1/5		1/1
93	GAPDH	NM_002046.4	Homo sapiens glyceraldehyde-3-phosphate dehydrogenase (GAPDH), transcript variant 1, mRNA.	1401	415	OK	OK	1/8	NM_001256799	2/2
94	TBP	NM_003194	Homo sapiens TATA box binding protein (TBP), mRNA.	1921	954	OK	OK	1/7	NM_001172085	2/2
95	B2M	NM_004048	Homo sapiens beta-2-microglobulin (B2M), mRNA.	987	122	OK	OK	1/3		1/1
96	PPIA	NM_021130	Homo sapiens peptidylprolyl isomerase A (cyclophilin A) (PPIA), mRNA.	2276	522	NG	OK	1/4		1/3

Biology Open • Supplementary information

Table S2. Top 10 gene categories of the up- or down-regulated genes in hiPS-hep compared to undifferentiated iPSCs

up-regulated gene in hiPS-hep

Category	*P*-value
Lipid Metabolism	1.3E-28
Metabolic Disease	6.53E-23
Vitamin and Mineral Metabolism	6.53E-23
Inflammatory Response	6.53E-23
Gastrointestinal Disease	8.32E-19
Cellular Movement	3.15E-17
Digestive System Development and Function	4.37E-16
Cell Death and Survival	2.27E-14
Carbohydrate Metabolism	1.03E-13
Protein Synthesis	1.49E-13

down-regulated gene in hiPS-hep

Category	*P*-value
Cancer	1.02E-19
Cellular Development	1.42E-13
Cell Cycle	1.45E-13
Cellular Assembly and Organization	7.72E-12
DNA Replication, Recombination, and Repair	6.38E-11
Cell Morphology	2.29E-10
Tissue Development	8.23E-10
Cell-To-Cell Signaling and Interaction	9.23E-10
Cellular Function and Maintenance	3.26E-09
Organismal Survival	9.43E-09

Biology Open • Supplementary information

Table S3. Top 100 up-regulated genes in hiPS-hep compared to undifferentiated hiPSCs

Gene Name	Fold Change	Gene Name	Fold Change
albumin	17.8	tryptophan 2,3-dioxygenase	12
haptoglobin-related protein	16.7	cytochrome P450 family 3 subfamily A member 5	11.9
aldolase, fructose-bisphosphate B	16.2	annexin A8 like 1	11.9
apolipoprotein H	15.8	arylacetamide deacetylase	11.8
apolipoprotein C3	15.8	fibrinogen beta chain	11.8
serum amyloid A1	15.6	glutathione S-transferase alpha 2	11.8
haptoglobin	15.5	ubiquitin D	11.8
fibrinogen like 1	14.7	alpha-2-macroglobulin	11.7
serpin family A member 3	14.7	vanin 1	11.7
transmembrane 4 L six family member 4	14.2	inter-alpha-trypsin inhibitor heavy chain 1	11.7
apolipoprotein C2	14.1	complement factor H	11.7
defensin beta 1	14	arginase 1	11.7
inter-alpha-trypsin inhibitor heavy chain 3	13.9	N-acetyltransferase 8 (putative)	11.6
serum amyloid A4, constitutive	13.9	hydroxyacid oxidase 1	11.6
serum amyloid A2	13.9	UDP glucuronosyltransferase family 1 member A10	11.5
aldo-keto reductase family 1 member B10	13.6	aldo-keto reductase family 1 member C2	11.5
nicotinamide N-methyltransferase	13.6	histidine rich glycoprotein	11.5
serpin family A member 1	13.5	coagulation factor II, thrombin	11.5
alcohol dehydrogenase 1C (class I), gamma polypeptid	13.2	HNF1 homeobox A	11.4
galectin 4	13.2	hydroxysteroid 17-beta dehydrogenase 2	11.4
transmembrane 4 L six family member 5	13	orosomucoid 1	11.4
serpin family C member 1	13	complement component 4 binding protein beta	11.4
hydroxysteroid 11-beta dehydrogenase 1	12.8	alpha 2-HS glycoprotein	11.4
retinol binding protein 4	12.7	complement factor I	11.3
carboxylesterase 1	12.7	caspase 1	11.3
cytochrome P450 family 8 subfamily B member 1	12.6	fibrinogen alpha chain	11.3
GC, vitamin D binding protein	12.6	vitronectin	11.3
fatty acid binding protein 1	12.5	sulfotransferase family 2A member 1	11.3
angiotensinogen	12.5	solute carrier family 40 member 1	11.2
aldo-keto reductase family 1 member B15	12.5	apolipoprotein B	11.2
acyl-CoA synthetase medium chain family member 2E	12.5	aldehyde dehydrogenase 1 family member A1	11.2
UDP glucuronosyltransferase family 2 member B7	12.4	flavin containing monooxygenase 3	11.2
apolipoprotein A1	12.4	complement component 4 binding protein alpha	11.1
cytochrome P450 family 2 subfamily C member 9	12.4	hyaluronan binding protein 2	11
cytochrome P450 family 2 subfamily C member 18	12.3	baculoviral IAP repeat containing 3	10.9
cytochrome P450 family 3 subfamily A member 7	12.3	complement C8 alpha chain	10.9
UDP glucuronosyltransferase family 2 member B4	12.3	orosomucoid 2	10.8
aldo-keto reductase family 1 member C4	12.2	APOBEC1 complementation factor	10.8
UDP glucuronosyltransferase family 2 member B10	12.2	secretory leukocyte peptidase inhibitor	10.7
asialoglycoprotein receptor 2	12.2	lipopolysaccharide binding protein	10.7
fibrinogen gamma chain	12.2	transmembrane 4 L six family member 1	10.7
inter-alpha-trypsin inhibitor heavy chain 2	12.2	UDP glucuronosyltransferase family 2 member B15	10.7
glutathione S-transferase alpha 5	12.2	serpin family A member 4	10.6
transferrin	12.2	cadherin related family member 2	10.6
bile acid-CoA:amino acid N-acyltransferase	12.1	acyl-CoA synthetase long chain family member 5	10.6
4-hydroxyphenylpyruvate dioxygenase	12.1	transthyretin	10.6
UDP glucuronosyltransferase family 1 member A6	12.1	PDZK1 interacting protein 1	10.5
amyloid P component, serum	12.1	cytochrome P450 family 4 subfamily A member 11	10.5
plasminogen	12.1	ATP binding cassette subfamily C member 3	10.5
UDP glucuronosyltransferase family 2 member B11	12.1	hepcidin antimicrobial peptide	10.5
